# Synthesis and SAR of the antistaphylococcal natural product nematophin from *Xenorhabdus nematophila*

**DOI:** 10.3762/bjoc.15.47

**Published:** 2019-02-25

**Authors:** Frank Wesche, Hélène Adihou, Thomas A Wichelhaus, Helge B Bode

**Affiliations:** 1 Molekulare Biotechnologie, Goethe University Frankfurt, Max-von-Laue-Str. 9, D-60438 Frankfurt am Main, Germany; 2 present address: Respiratory, Inflammation and Autoimmunity, Innovative Medicines and Early Development, Biotech Unit, AstraZeneca, Pepparedsleden 1, Mölndal 43183, Sweden; 3 present address: Cardiovascular and Metabolic Diseases, Innovative Medicines and Early Development, Biotech Unit, AstraZeneca, Pepparedsleden 1, Mölndal 43183, Sweden; 4 present address: AstraZeneca MPI Satellite Unit, Abteilung Chemische Biologie, Max Planck Institut für Molekulare Physiologie, Otto-Hahn-Str. 11, 44227 Dortmund, Germany; 5 Institut für Medizinische Mikrobiologie und Krankenhaushygiene, Universitätsklinikum Frankfurt, Paul-Ehrlich-Str. 40, D-60596 Frankfurt am Main, Germany; 6 Buchmann Institute for Molecular Life Sciences (BMLS), Goethe University Frankfurt, Max-von-Laue-Str. 15, D-60438 Frankfurt am Main, Germany

**Keywords:** azaindole, fluorescent dye, MRSA, nematophin, *Staphylococcus aureus*

## Abstract

The repeated and improper use of antibiotics had led to an increased number of multiresistant bacteria. Therefore, new lead structures are needed. Here, the synthesis and an expanded structure–activity relationship of the simple and antistaphylococcal amide nematophin from *Xenorhabdus nematophila* and synthetic derivatives are described. Moreover, the synthesis of intrinsic fluorescent derivatives, incorporating azaindole moieties was achieved for the first time.

## Introduction

Microorganisms present a rich source of bioactive natural products of pharmacological importance against an emerging number of multiresistant bacteria [1]. Such examples are *Xenorhabdus* sp., Gram-negative entomopathogenic bacteria which live in symbiosis with soil-living nematodes of the genera *Steinernema* [2–3]. During a complex life cycle the nematode–bacteria pair infects and kills insect larvae, whereby *Xenorhabdus* produce a broad range of natural products with antimicrobial properties [4–8]. As the *Steinernema–Xenorhabdus* complex is not pathogenic against humans, they are widely used as biocontrol agents in agriculture [9].

Natural products produced by bacteria play an important role in the bacteria/nematode/insect life cycle and most natural products are non-ribosomal peptides (NRP), e.g., rhabdopeptides [10–11] and polyketide–NRP hybrids, like xenocoumacins [12–13]. The quite simple amide nematophin (**1**) is another well-known member of natural products common in all *Xenorhabdus nematophila* strains and was first described by Li et al. in 1997 for their antimicrobial properties [14]. Simply, **1** is the condensation product of 3-methyl-2-oxopentanoic acid and tryptamine and showed good activities against different Gram-positive pathogens like *Staphylococcus aureus*, including methicillin-resistant *S. aureus* (MRSA) comparable with activities of vancomycin. Recently, the biosynthesis of **1** was elucidated by our group as well as the 2-phenylethylamine derivative **2** with an α-keto amide moiety, which could be identified upon heterologous expression of the appropriate gene cluster in *E. coli* [15]. Moreover, elongated nematophin derivatives, namely nevaltophins from *Xenorhabdus* PB62.4, were described incorporating an additional valine. As **1** and nevaltophines act as prophenoloxidase activators, it is suggested that they have a specific role in the bacteria/nematode/insect symbiosis.

Little or nothing is known about the mode of action of this simple amide against *S. aureus*. Structure–bioactivity studies revealed that the α-keto moiety and the amide moiety itself are required for bioactivity [16]. These findings suggest a specific interaction of this electrophilic moiety with a nucleophile. Such an interaction was previously reported for the macrocyclic peptide cyclotheonamide A, isolated from marine sponge *Theonella* sp. Cyclotheonamide A is described as a potent inhibitor of various proteases, in particular trypsin and thrombin [17–19]. Hereby, the α-keto amide covalently binds to the serine oxygen in the active site under formation of a stable tetrahedral hemiketal. Furthermore, substitution of the indole hydrogen by alkyl, aryl or benzyl improves the in vitro antistaphylococcal activity. In contrast, the incorporation of smaller heterocycles like pyridine and imidazole as well as isosteric benzimidazole instead of the indole moieties lead to a loss of antibacterial activity. Kennedy et al. could synthesize a 2-phenyl derivative that showed nanomolar activity against *S. aureus* [20]. To the detriment of this compound class, all derivatives lose their antibacterial activity in the presence of serum in vitro in serial broth and agar dilution method [16,20]. Even with the use of charged groups as modifiers, serum-protein binding could not be avoided. However, we were interested in expanding the aforementioned structure–activity studies regarding the substitution of the indole moiety by different aromatic systems as well as substitution of side chains in the α-keto carboxylic acids to generate more derivatives of this fascinating small and bioactive amide.

## Results and Discussion

**Scheme 1:**
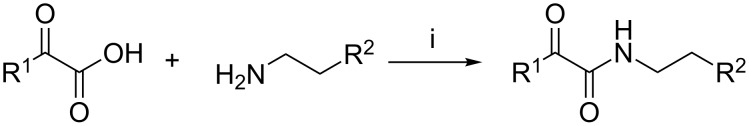
General procedure for the synthesis of nematophin and related derivatives. i) 1.5 equiv α-keto carboxylic acid, 1.0 equiv amine, 1.5 equiv EDC·HCl, 1.5 equiv HOBt, 2.0 equiv DIPEA in DMF (*c* 0.1 M), 25 W, 75 °C, 20 min.

We first synthesized **1** and 1-methylnematophin (**3**) as standards to confirm preliminary results. We then initiated the synthesis of derivatives **2**, and **4**–**12**. Briefly, the appropriate α-keto carboxylic acid was coupled to the respective amine. Amide bond formation was achieved using 1-ethyl-3-(3-dimethylaminopropyl)carbodiimide hydrochloride (EDC·HCl), 1-hydroxybenzotriazole (HOBt) and *N*,*N*-diisopropylethylamine (DIPEA) in DMF under microwave irradiation (Scheme 1). Racemic **1** was synthesized using tryptamine and (*rac*)-3-methyl-2-oxopentanoic acid. Similarly, compounds **2**–**7** were synthesized using different amines including 2-phenylethylamine (for **2**), 1-methyltryptamine (for **3**), 2-(2-naphthyl)ethylamine hydrochloride (for **4**), 2-(1-naphthyl)ethylamine hydrochloride (for **5**), 2-(1*H*-inden-3-yl)ethylamine hydrobromide (for **6**) [21], and 2-(1-benzofuran-3-yl)ethylamine (for **7**), respectively. Compounds **8**–**12** were synthesized by coupling tryptamine with different α-keto carboxylic acids, including 3-furylglyoxylic acid (for **8**), 3-indoleglyoxylic acid (for **9**), phenylglyoxylic acid (for **10**), and isomeric 3-methylpent-2-enoic acid (for **11** and **12**) [22], respectively. Compounds **11** and **12** were separated during the isolation process and initially constructed to enable target identification. Compounds **11** and **12** might act as Michael acceptor (α,β-unsaturated carbonyl) and attach irreversibly and covalently to a potential target [23]. After purification and characterization, the above mentioned compounds were tested against different pathogenic strains, i.e., methicillin-susceptible *S. aureus* (MSSA), methicillin-resistant *S. aureus* (MRSA), *Enterococcus faecalis*, and *Micrococcus luteus*. The yields and bioactivities are summarized in Table 1 (and Table S1, Supporting Information File 1).

**Table 1:** Summary of synthesized nematophin derivatives (**1**–**12**) and their bioactivity (MIC in µg/mL) against *S. aureus* (MSSA and MRSA).

		MIC (µg/mL)^b^	
structure	yield^a^	*S. aureus* (MSSA) ATCC 29213	MRSA (MRSA) ATCC 43300

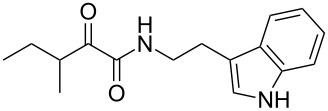 **1**	46%	0.5	0.5

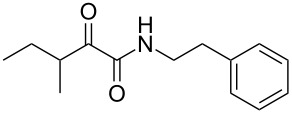 **2**	56%	16	32

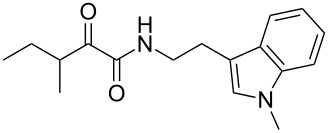 **3**	74%	0.125	0.031

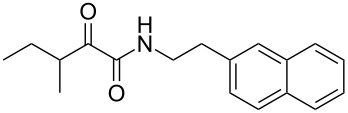 **4**	48%	2	4

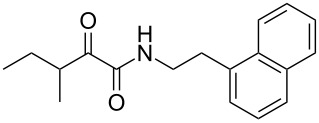 **5**	70%	1	4

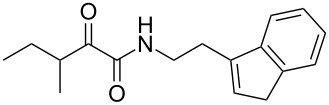 **6**	26%	1	1

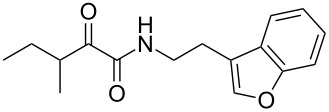 **7**	54%	>128	>128

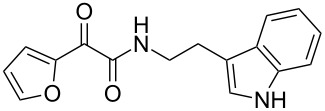 **8**	59%	64	>64

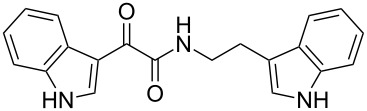 **9**	84%	>64	>64

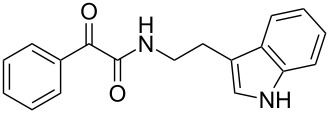 **10**	47%	8	8

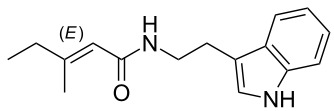 **11**	59%^c^	>128	>128

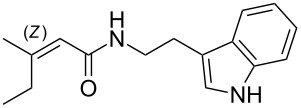 **12**	59%^c^	>128	>128

^a^Purification using column chromatography on silica gel. ^b^Median of three experiments. ^c^Derivatives were synthesized using an isomeric mixture of 3-methylpent-2-enoic acid.

As previously described, the minimum inhibitory concentration (MIC) of **1** improves by alkylation of the indole moiety as seen for **3** [16] even **1** and **3** show a good activity against MRSA. Although for all other tested compounds no better activity than that of the original natural product **1** was observed, **6** showed an activity comparable to **1** against MRSA. Since only a slight activity could be observed for some compounds against additionally tested bacteria, i.e., *M. luteus* ATCC 9431 and *E. faecalis* ATCC 29212, a staphylococcal-specific target is suggested. All derivatives with α-keto-β-methylvaleric moiety (**1**–**7**) were active against *S. aureus*. Substitution of the side chain in the α-keto amide significantly affects the bioactivity, as seen for compounds **8**, **9** and **10**. Furthermore, attempts to identify a possible target by the incorporation of an α,β-unsaturated carbonyl moiety, as it should bind irreversible to a potential target, instead of the α-keto amide in **11** and **12** are stalled as it led to a complete loss of bioactivity.

To the best of our knowledge, no one has ever tried to incorporate azaindole moieties in small natural products and tried to use them for fluorescence imaging. This approach may circumvent the above-mentioned impasse and should allow at least localization studies in *S. aureus* cells. Azaindoles are isosteric to indole, whereas one of the endocyclic methines is substituted with nitrogen, and thus leads to an increased and red-shifted fluorescence [24–26]. Budisa and co-workers have already used azatryptophans to study proteins with intrinsic green and blue fluorescence [27–28]. The azaindole moiety allows a linker-less incorporation of a fluorescent label with minimal disturbance.

**Scheme 2:**
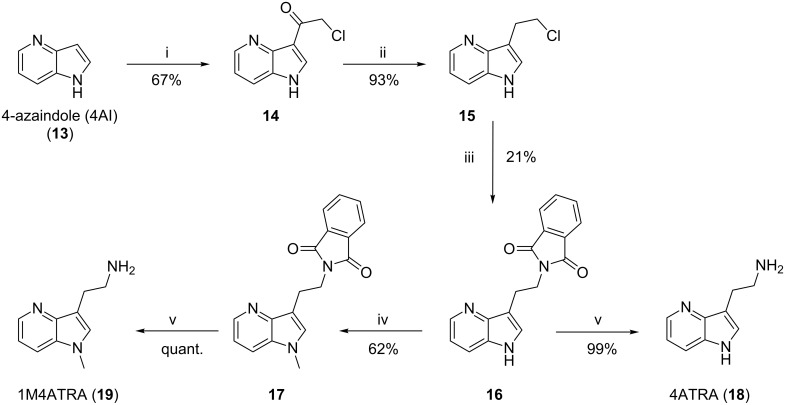
Synthesis of azatryptamines (4-azatryptamine (4ATRA) and 1-methyl-4-azatryptamine (1M4ATRA)). i) 5.0 equiv AlCl_3_, 5.0 equiv chloroacetyl chloride in DCM, overnight, rt 67%; ii) 7.0 equiv Et_3_SiH in TFA, overnight, rt, 93%; iii) 1.1 equiv potassium phthalimide, in DMF, 100 °C, 21%; iv) 1.2 equiv NaH, 1.0 equiv MeI in DMF, overnight, rt, 62%; v) 5.0 equiv N_2_H_4_·H_2_O in EtOH, 90 °C, 2 h, 25 W, 99% to quant.

**Scheme 3:**
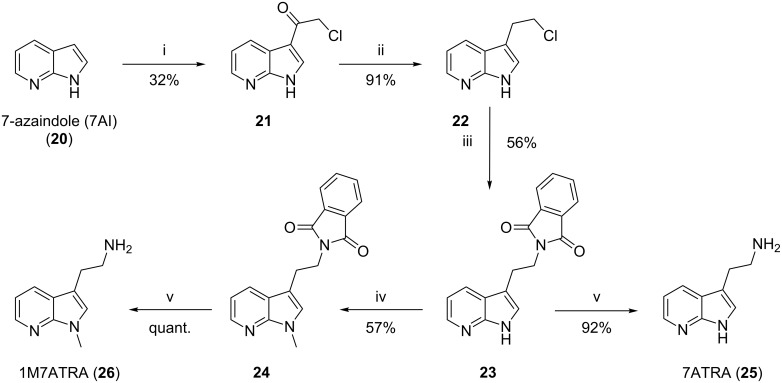
Synthesis of azatryptamines (7-azatryptamine (7ATRA) and 1-methyl-7-azatryptamine (1M7ATRA)). i) 5.0 equiv AlCl_3_, 5.0 equiv ClCH_2_COCl in DCM, overnight, rt 32%; ii) 7.0 equiv Et_3_SiH in TFA, overnight, rt, 93%; iii) 1.1 equiv potassium phthalimide, in DMF, 100 °C, 56%; iv) 1.2 equiv NaH, 1.0 equiv MeI in DMF, overnight, rt, 57%; v) 5.0 equiv N_2_H_4_·H_2_O in EtOH, 2 h, 90 °C, 25 W, 92% to quant.

Therefore, four fluorescent derivatives of nematophin were designed and their synthesis initiated. The syntheses of the appropriate azatryptamine derivatives (**17**, **18**, **25**, and **26**) were achieved from the non-expensive and commercially available 4- and 7-azaindole (**13** and **20**), respectively. First, **13** and **20** were converted in a Friedel–Crafts acylation with chloroacetyl chloride (ClCH_2_COCl) and aluminium chloride (AlCl_3_) in DCM to give compounds **14** and **21**. Subsequent reduction was achieved with triethylsilane (Et_3_SiH) in TFA to give **15** and **22**. For the synthesis of the primary amine, halides **15** and **22** were converted in a Gabriel synthesis with potassium phthalimide in DMF to the appropriate phthalimides **16** and **23** [29]. These intermediary compounds **16** and **23** also allowed an *N*-methylation of the azaindole moiety with sodium hydride (NaH) and methyl iodide (MeI) to yield **17** and **24**. By ethanolic hydrazinolysis and microwave irradiation the phthalimides (**16**, **17**, **23**, and **24**) were deprotected yielding the desired primary amines in good yields (**18**, **19**, **25**, and **26**) (Scheme 2 and Scheme 3). With all building blocks available, the synthesis of the appropriate fluorescent derivatives was performed as before, using (*rac*)-3-methyl-2-oxopentanoic acid. Yields after coupling and bioactivities are summarized in Table 2.

**Table 2:** Summary of synthesized nematophin derivatives (**27**–**30**) and their bioactivity (MIC in µg/mL) against *S. aureus* (MSSA and MRSA).

		MIC (µg/mL)^b^	
structure	yield^a^	*S. aureus* (MSSA) ATCC 29213	MRSA (MRSA) ATCC 43300

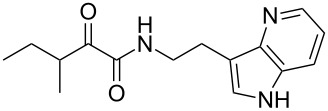 **27**	32%	32	64

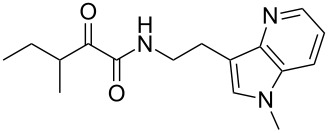 **28**	16%	16	>128

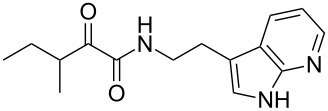 **29**	72%	16	64

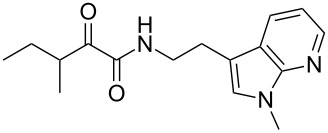 **30**	28%	4	32

^a^Purification using column chromatography on silica gel. ^b^Median of three experiments.

Whereas the biological data, for example against *S. aureus* or MRSA shows a decreased activity for all azaindole derivatives (compare **1** with **27** or **28**) as observed for isosteric benzimidazoles [20], an increased activity for all methylated derivatives (compare pairs of **1**/**3**, **27**/**28**, and **29**/**30**) could be observed for at least MSSA. This observation may correlate to the molecular polarity, and thus hydrophilicity of the cyclic moiety, whereas almost all hydrophobic moieties showed good antistaphylococcal activity as seen for naphthyl (**4** and **5**) and indene (**6**). However, the increase of hydrophobicity influences the passive membrane diffusion and therefore might influence how the compounds get to their actual target [30]. Moreover, a specific minimal size of the cyclic moiety must be fulfilled as, e.g., **2** with a phenyl moiety is less active in vitro.

## Conclusion

Despite their lower bioactivities, the azaindole derivatives are currently under investigation upon their use for fluorescence imaging. Furthermore, derivatives **3** and **6** will be further studied for identification of their actual target in *S. aureus*. This could be a serine protease that can also be addressed in the future by more stable compounds. Moreover, it might be possible to use nematophin and its derivatives for topical treatment of *S. aureus* infections.

## Supporting Information

File 1 General procedure, supplemantary table and NMR spectra.
