# Supplementary material for: Synthesis and SAR of the antistaphylococcal natural product nematophin from Xenorhabdus nematophila
Source: Beilstein J Org Chem. 2019 Feb 25;15:535–41. doi: 10.3762/bjoc.15.47 (PMC6404511; doi:10.3762/bjoc.15.47)
Supplement: File 1 — General procedure, supplemantary table and NMR spectra. [file Beilstein_J_Org_Chem-15-535-s001.pdf]

BEILSTEIN JOURNAL OF ORGANIC CHEMISTRY

Supporting Information

for

Synthesis and SAR of the antistaphylococcal natural product nematophin from *Xenorhabdus nematophila*

Frank Wesche, Hélène Adihou, Thomas A. Wichelhaus and Helge B. Bode

Beilstein J. Org. Chem. **2019,** *15,* 535–541. doi:10.3762/bjoc.15.47

General procedure, supplemantary table and NMR spectra

License and Terms: This is a supporting information file under the terms of the Creative Commons Attribution License (http://creativecommons.org/licenses/by/4.0). Please note that the reuse, redistribution and reproduction in particular requires that the authors and source are credited.
The license is subject to the *Beilstein Journal of Organic Chemistry* terms and conditions: (https://www.beilstein-journals.org/bjoc)

Content

General procedures **S2**
Supplementary table **S21**
NMR Spectra **S22**

General procedures

All reactions were carried out under an atmosphere of nitrogen and with dry solvents, unless otherwise stated. Dry solvents and chemicals were purchased from Sigma-Aldrich (Germany), Acros Organics (Belgium), Alfa Aesar (Germany), and Merck Millipore (Germany) in highest commercial available purity and were used without further purification.

NMR

The ^{1}H and ^{13}C spectra were recorded on an AV-250, AV-400- or AV-500-spectrometer from Bruker (USA). All measurements were performed at room temperature and chemical shifts are given in ppm (parts per million) compared to tetramethylsilane [δ = 0 ppm], referring on second internal standard. To explain multiplicities the following abbreviations were used: s = singlet, d = doublet, t = triplet, q = quartet, m = multiplet.

HPLC-HRMS

5 µl of the sample (dissolved crude or purified compound in MeOH, H_2O, ACN or mixture of those) were injected and analysed via HPLC-ESI/MS by a Dionex UltiMate 3000 system coupled to a Bruker Impact II QTOF with a ACQUITY UPLC™ BEH C18 column (130 Å, 2.1 mm × 100 mm, 1.7 µm particle size, Waters) at a flow rate of 0.4 mL/min for 16 min and 40 °C, using ACN and water supplemented with 0.1% formic acid (v/v) in a gradient ranging from 5 to 95% ACN. For detection positive mode with a scanning range from 100–1200 *m*/*z* was used. HR-ESI-MS analysis was carried out by using an internal calibrant (10 mM sodium formate solution) and the same linear gradient from 5 to 95% ACN (0.1% formic acid).

TLC

For thin-layer chromatography (TLC) Polygram SIL G/UV254- sheets from Macherey-Nagel (Germany) were used. The detection of the substances was achieved by exposure to a UV-lamp (254 nm) or through staining with potassium permanganate (3 g $KMnO_4$, 5 g Na_2CO_3 in 250 mL water).

Column chromatography

The separation and purification of the crude products was achieved with a column chromatographic SP1 systems form Biotage (Sweden) and prepacked columns. The used solvent mixtures of *n*-hexane and EtOAc, $CHCl_3$ or DCM and MeOH were tested previously by TLC. Different flow rates of 15 mL/min, 30 mL/min, and 30 mL/min, where used for different columns of SNAP KP-Sil® 10 g, 25 g and 50 g, respectively. After separation, collected fractions were combined according to the curve of the UV-chromatogram, and after retention factors from the TLC, respectively.

Minimum inhibitory concentration (MIC) determination

Minimal inhibitory concentrations (MICs) of nematophin and its derivatives were determined according to microdilution method established by Clinical and Laboratory Standards Institute (CLSI, Methods for dilution antimicrobial susceptibility tests for bacteria that grow aerobically; approved standard 7th ed. Document M7-A7. Wayne, PA; 2006) for bacterial strains *Staphylococcus aureus* (ATCC29213), MRSA (ATCC43300), *Micrococcus luteus* (ATCC9341), and *Enterococcus faecalis* (ATCC29212). Briefly, 95 µL of CAMHB (cation-adjusted Mueller-Hinton broth) medium supplemented with the different compounds at various concentrations ranging from 128 to 0.06 µg/mL were added to each well of a 96-well microtiter plate. 5 µL of a bacterial suspension were added to each well to yield a final concentration of about 5×10^5 cells/mL. The MIC was defined as the lowest concentration of antimicrobial agent that completely inhibited growth of the organism in a microdilution well as detected by the unaided eye. For quality control and to monitor for accuracy the gentamicin MIC was verified to fall within the acceptable limits. .

Synthesis of nematophin and derivatives

General procedure for synthesis of nematophin and related derivatives

To a mixture of α-keto carboxylic acid (1.5 equiv), EDC·HCl (1.5 equiv), HOBt (1.5 equiv) and DIPEA (2.0 equiv) in DMF (c = 0.1 M) was added the appropriate amine (1.0 equiv) and reaction was conducted under microwave irradiation at 75 °C, and 25 W for 20 min (Discover system, CEM Corporation/ USA). Afterwards saturated aqueous $NaHCO_3$ was added and extracted three times with EtOAc. The combined organic layers were dried over $MgSO_4$, concentrated and purified by flash chromatography (Biotage/ Sweden, SNAP KP-Sil®, 10 g, EtOAc in *n*-hexane or MeOH in DCM as eluent).

Synthesis of 4- and 7-azatryptamine (18 and 25) and 1-methyl-4- and 1-methyl-7-azatryptamine (19 and 26)

First two steps of the synthesis were performed slightly modified as described previously.[1]

i) Synthesis of 2-chloro-1-(1*H*-pyrrolo[3,2-*b*]pyridin-3-yl)ethanone (14) and 2-chloro-1-(1*H*-pyrrolo[2,3-*b*]pyridin-3-yl)ethanone (21)

Under an atmosphere of nitrogen, a suspension of azaindole (1.0 equiv) and $AlCl_3$ (5 equiv) was stirred in dry DCM (c = 0.2 M) for 1 h. Afterwards a solution of chloroacetyl chloride (5 eq.) in dry DCM (c = 2.5 M) was added slowly and the mixture was stirred at rt overnight. The solvent was removed and the residue was dissolved in water and basified carefully with saturated Na_2CO_3 solution until pH 9 and extracted three times with EtOAc. The combined organic layers were dried over $MgSO_4$ and the solvent was removed under reduced pressure to give title compounds as slightly yellow solids.

ii) Synthesis of 3-(2-chloroethyl)-1*H*-pyrrolo[3,2-*b*]pyridine (15) and 3-(2-chloroethyl)-1*H*-pyrrolo[2,3-*b*]pyridine (22)

14 or **21** (1 equiv) was dissolved in TFA (c = 0.2 M) and triethylsilane (7.0 equiv) was added. The resulting mixture was stirred at rt overnight. Then EtOAc was added and the solution basified with saturated Na_2CO_3 until pH > 8. The phases were separated and the aqueous layer was extracted two times with EtOAc. The combined organic

layers were dried over $MgSO_4$ and the solvent was removed under reduced pressure. The crude product was purified by flash chromatography (SNAP KP-Sil®, 25 g, 10 % → 100 % EtOAc in *n*-hexane) to give title compounds as slightly yellow solids.

iii) Synthesis of 2-(2-(1*H*-pyrrolo[3,2-*b*]pyridin-3-yl)ethyl)isoindoline -1,3-dione (16) and 2-(2-(1*H*-pyrrolo[2,3-*b*]pyridin-3-yl)ethyl)isoindoline-1,3-dione (23)

A mixture of potassium phthalimide (1.1 equiv) and educts **15** or **22** (1.0 equiv) in DMF (*c* = 0.1 M) were heated for 2 h at 100 °C. Then the solvent was removed and ice water was added and extracted three times with DCM. The combined organic layers were dried over $MgSO_4$, the solvent was removed under reduced pressure and the crude product was purified by flash-chromatography (SNAP KP-Sil®, 25 g, 10 % → 100 % EtOAc in *n*-hexane) to give title compounds as slightly yellow solids.

iv) Synthesis of 2-(2-(1-methyl-1*H*-pyrrolo[3,2-*b*]pyridin-3-yl)ethyl)isoindoline-1,3-dione (17) and 2-(2-(1-methyl-1*H*-pyrrolo[2,3-*b*]pyridin-3-yl)ethyl)isoindoline-1,3-dione (24)

To a mixture of sodium hydride (60% suspension in mineral oil, 1.2 equiv) in DMF (*c* = 0.1 M), a solution of educts **16** or **23** (1.0 equiv) in DMF (*c* = 0.1 M) was slowly added. Afterwards MeI (1.0 equiv) was added and the reaction mixture was stirred at room temperature overnight. The mixture was concentrated by half under reduced pressure and ice water was added. After triple extraction with EtOAc, the combined organic layers were dried over $MgSO_4$ and the solvent was removed under reduced pressure. Purification by flash-chromatography (SNAP KP-Sil®, 10 g, 20% → 100% EtOAc in *n*-hexane) afforded title compounds as pale-yellow solids.

v) Deprotection of phthalimide – synthesis of 2-(1*H*-pyrrolo[3,2-*b*]pyridin-3-yl)ethylamine (18), 2-(1-methyl-1*H*-pyrrolo[3,2-*b*]pyridin-3-yl)ethylamine (19), 2-(1*H*-pyrrolo[2,3-*b*]pyridin-3-yl)ethylamine (25) and 2-(1-methyl-1*H*-pyrrolo[2,3-*b*]pyridin-3-yl)ethylamine (26)

Phthalimide (**16**, **17**, **23** or **24**) was dissolved in ethanol (*c* = 35 mM) and 5.0 eq. of 50–60% hydrazine hydrate (Sigma-Aldrich, Germany) solution was added. The mixture was heated up in a microwave for 2 h at 90 °C and 25 W. Afterwards the

precipitated phthalic acid was removed by filtration and 1 M NaOH was added. The aqueous phase was extracted at least three times with chloroform/isopropanol (4:1) (v/v) and the combined organic phases were dried over $MgSO_4$. Solvent was removed under reduced pressure to give the azatryptamines.

Nematophin - *N*-(2-(1*H*-Indol-3-yl)ethyl)-3-methyl-2-oxopentanamide (1)

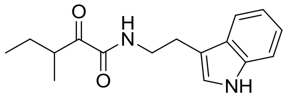

1

Yield: 46% (27.9 mg)

HRMS (*m/z*): calc. for $C_{16}H_{20}N_2O_2$ $[M+H]^+$: 273.1598; found: 273.1595.

^{1}H NMR (400 MHz, Chloroform-*d*) δ 8.18 (s, 1H), 7.61 (d, *J* = 7.8 Hz, 1H), 7.37 (d, *J* = 8.2 Hz, 1H), 7.25 – 7.18 (m, 1H), 7.17 – 7.05 (m, 2H), 7.02 (d, *J* = 2.4 Hz, 1H), 3.65 (q, *J* = 6.7 Hz, 2H), 3.55 – 3,47 (m, 1H), 3.03 (t, *J* = 6.9 Hz, 2H), 1.73 (tt, *J* = 13.6, 7.4 Hz, 1H), 1.47 – 1.33 (m, 1H), 1.10 (d, *J* = 7.0 Hz, 3H), 0.90 (t, *J* = 7.4 Hz, 3H).

^{13}C NMR (101 MHz, Chloroform-*d*) δ 202.5, 160.2, 136.6, 127.3, 122.4, 122.2, 119.7, 118.7, 112.6, 111.4, 40.5, 39.7, 25.6, 25.3, 15.3, 11.6.

3-Methyl-2-oxo-*N*-phenethylpentanamide (2)

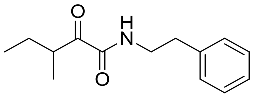

2

Yield: 56% (52.7 mg)

HRMS (*m/z*): calc. for $C_{14}H_{19}NO_2$ $[M+H]^+$: 234.1489; found: 234.1487.

^{1}H NMR (500 MHz, Chloroform-*d*) δ 7.31 (t, *J* = 7.4 Hz, 2H), 7.25 – 7.22 (m, 1H), 7.21 – 7.17 (m, 2H), 6.99 (s, 1H), 3.60 – 3.53 (m, 2H), 3.52 – 3.44 (m, 1H), 2.86 (t, *J* = 7.1 Hz, 2H), 1.76 – 1.67 (m, 1H), 1.45 – 1.34 (m, 1H), 1.08 (d, *J* = 7.0 Hz, 3H), 0.88 (t, *J* = 7.5 Hz, 3H).

^{13}C NMR (126 MHz, Chloroform-*d*) δ 202.4, 160.1, 138.4, 128.9, 128.8, 126.9, 40.6, 40.5, 35.6, 25.6, 15.3, 11.6.

1-Methylnematophin - 3-Methyl-*N*-(2-(1-methyl-1*H*-indol-3-yl)ethyl)-2-oxopentanamide (3)

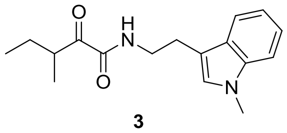

3

Yield: 74% (85.0 mg)

HRMS (*m/z*): calc. for $C_{17}H_{22}N_2O_2$ $[M+H]^+$: 287.1754; found: 287.1753.

^{1}H NMR (500 MHz, Chloroform-*d*) δ 7.59 (d, *J* = 7.9 Hz, 1H), 7.31 (d, *J* = 8.3 Hz, 1H), 7.26 – 7.22 (m, 1H), 7.15 – 7.09 (m, 1H), 7.06 (s, 1H), 6.89 (s, 1H), 3.76 (s, 3H), 3.62 (q, *J* = 6.9 Hz, 2H), 3.50 (h, *J* = 6.9 Hz, 2H), 3.00 (t, *J* = 6.9 Hz, 1H), 1.72 (tt, *J* = 13.7, 7.5 Hz, 1H), 1.44 – 1.35 (m, 1H), 1.09 (d, *J* = 6.9 Hz, 3H), 0.89 (t, *J* = 7.5 Hz, 3H).

^{13}C NMR (126 MHz, Chloroform-*d*) δ 202.5, 160.1, 137.3, 127.7, 127.0, 122.0, 119.2, 118.9, 111.0, 109.5, 40.5, 39.8, 32.8, 25.6, 25.2, 15.3, 11.7.

3-Methyl-*N*-(2-(naphthalen-2-yl)ethyl)-2-oxopentanamide (4)

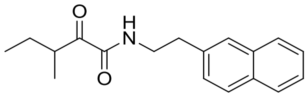

4

Yield: 48% (54.4 mg)

HRMS (*m/z*): calc. for $C_{18}H_{21}NO_2$ $[M+H]^+$: 284.1645; found: 284.1642.

^{1}H NMR (500 MHz, Chloroform-*d*) δ 7.87 – 7.74 (m, 3H), 7.64 (s, 1H), 7.51 – 7.41 (m, 2H), 7.33 (dd, *J* = 8.4, 1.8 Hz, 1H), 7.03 (s, 1H), 3.66 (q, *J* = 7.0 Hz, 2H), 3.48 (h, *J* = 6.9 Hz, 1H), 3.02 (t, *J* = 7.1 Hz, 2H), 1.75 – 1.64 (m, 1H), 1.43 – 1.32 (m, 1H), 1.07 (d, *J* = 7.0 Hz, 3H), 0.87 (t, *J* = 7.5 Hz, 3H).

^{13}C NMR (126 MHz, Chloroform-*d*) δ 202.4, 160.2, 135.8, 133.7, 132.5, 128.6, 127.8, 127.6, 127.3, 127.1, 126.3, 125.8, 40.6, 40.5, 35.7, 25.6, 15.2, 11.6.

3-Methyl-*N*-(2-(naphthalen-1-yl)ethyl)-2-oxopentanamide (5)

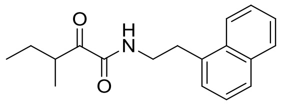

5

Yield: 70% (79.0 mg)

HRMS (*m/z*): calc. for $C_{18}H_{21}NO_2$ $[M+H]^+$: 284.1645; found: 284.1642.

^{1}H NMR (500 MHz, Chloroform-*d*) δ 8.09 (d, *J* = 8.3 Hz, 1H), 7.87 (d, *J* = 8.8 Hz, 1H), 7.77 (d, *J* = 8.1 Hz, 1H), 7.57 – 7.47 (m, 2H), 7.41 (dd, *J* = 8.2, 7.0 Hz, 1H), 7.32 (d, *J* = 7.0 Hz, 1H), 7.08 (s, 1H), 3.72 – 3.66 (m, 2H), 3.51 (h, *J* = 6.8 Hz, 1H), 3.34 (t, *J* = 7.3 Hz, 2H), 1.78 – 1.68 (m, 1H), 1.45 – 1.36 (m, 1H), 1.10 (d, *J* = 6.9 Hz, 3H), 0.90 (t, *J* = 7.5 Hz, 3H).

^{13}C NMR (126 MHz, Chloroform-*d*) δ 202.4, 160.3, 134.4, 134.1, 131.9, 129.1, 127.7, 126.9, 126.5, 125.9, 125.7, 123.6, 40.5, 40.1, 32.8, 25.6, 15.3, 11.6.

***N*-(2-(1*H*-Inden-3-yl)ethyl)-3-methyl-2-oxopentanamide (6)**

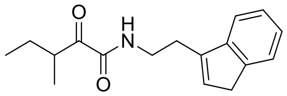

6

Yield: 26% (28.4 mg)

HRMS (*m/z*): calc. for $C_{17}H_{21}NO_2$ $[M+H]^+$: 272.1645; found: 272.1643.

^{1}H NMR (500 MHz, Chloroform-*d*) δ 7.48 (d, *J* = 7.3 Hz, 1H), 7.38 (d, *J* = 7.5 Hz, 1H), 7.34 – 7.28 (m, 1H), 7.23 (td, *J* = 7.4, 1.2 Hz, 1H), 7.08 (s, 1H), 6.30 (t, *J* = 1.8 Hz, 1H), 3.68 – 3.63 (m, 2H), 3.50 (q, *J* = 6.8 Hz, 1H), 3.37 (t, *J* = 2.0 Hz, 2H), 2.86 – 2.80 (m, 2H), 1.77 – 1.68 (m, 1H), 1.46 – 1.37 (m, 1H), 1.10 (d, *J* = 6.9 Hz, 3H), 0.89 (t, *J* = 7.5 Hz, 3H).

^{13}C NMR (126 MHz, Chloroform-*d*) δ 202.4, 160.1, 144.7, 144.5, 140.9, 129.9, 126.4, 125.1, 124.1, 119.0, 40.5, 38.1, 37.9, 27.7, 25.6, 15.3, 11.6.

_N_-(2-(Benzofuran-3-yl)ethyl)-3-methyl-2-oxopentanamide (7)

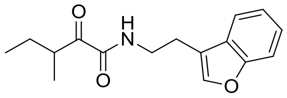

7

Yield: 54% (59.4 mg)

HRMS (_m/z_): calc. for $C_{16}H_{19}NO_3$ $[M+H]^+$: 274.1438; found: 274.1436.

^{1}H NMR (500 MHz, Chloroform-_d_) δ 7.57 (d, _J_ = 7.2 Hz, 1H), 7.51 – 7.44 (m, 2H), 7.34 – 7.28 (m, 1H), 7.28 – 7.23 (m, 1H), 7.09 (s, 1H), 3.65 (q, _J_ = 7.0 Hz, 2H), 3.53 – 3.45 (m, 1H), 2.95 (t, _J_ = 7.3 Hz, 2H), 1.77 – 1.67 (m, 1H), 1.45 – 1.35 (m, 1H), 1.09 (d, _J_ = 7.0 Hz, 3H), 0.89 (t, _J_ = 7.5 Hz, 3H).

^{13}C NMR (126 MHz, Chloroform-_d_) δ 202.3, 160.2, 155.6, 142.0, 127.7, 124.7, 122.7, 119.5, 116.9, 111.8, 40.5, 38.9, 25.6, 23.8, 15.3, 11.6.

_N_-(2-(1_H_-Indol-3-yl)ethyl)-2-(furan-2-yl)-2-oxoacetamide (8)

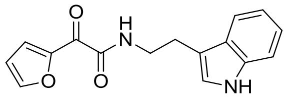

8

Yield: 59% (66.5 mg)

HRMS (_m/z_): calc. for $C_{16}H_{14}N_2O_3$ $[M+H]^+$: 283.1077; found: 283.1075.

^{1}H NMR (500 MHz, DMSO-d_6) δ 10.82 (s, 1H), 9.03 – 8.96 (m, 1H), 8.15 (dd, _J_ = 1.6, 0.7 Hz, 1H), 7.82 (dd, _J_ = 3.6, 0.7 Hz, 1H), 7.56 (d, _J_ = 7.8 Hz, 1H), 7.33 (d, _J_ = 8.1 Hz, 1H), 7.17 (d, _J_ = 2.2 Hz, 1H), 7.09 – 7.03 (m, 1H), 6.97 (ddd, _J_ = 7.9, 7.1, 0.9 Hz, 1H), 6.78 (dd, _J_ = 3.6, 1.7 Hz, 1H), 3.44 (q, 2H), 2.92 (t, _J_ = 7.5 Hz, 2H).

^{13}C NMR (126 MHz, DMSO-d_6) δ 175.3, 161.6, 150.3, , 149.3, 136.3, 127.3, 125.4, 122.8, 121.1, 118.4, 113.3, 111.5, 111.5, 39.5, 24.8.

***N*-(2-(1*H*-Indol-3-yl)ethyl)-2-(1H-indol-3-yl)-2-oxoacetamide (9)**

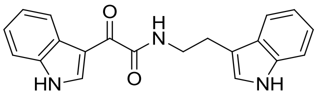

9

Yield: 84% (111.9 mg)

HRMS (*m/z*): calc. for $C_{20}H_{17}N_3O_2$ $[M+H]^+$: 332.1394; found: 332.1390.

^{1}H NMR (500 MHz, DMSO-*d*$_6$) δ 10.82 (s, 1H), 8.81 (t, *J* = 5.9 Hz, 1H), 8.74 (s, 1H), 8.24 – 8.19 (m, 1H), 7.34 (d, *J* = 8.1 Hz, 1H), 7.29 – 7.22 (m, 2H), 7.19 (d, *J* = 2.2 Hz, 1H), 7.09 – 7.04 (m, 1H), 7.02 – 6.96 (m, 1H), 3.53 – 3.50 (m, 3H), 2.95 (t, *J* = 7.5 Hz, 2H).

1H NMR (500 MHz, DMSO) δ 10.82 (s, 1H), 8.81 (t, J = 5.9 Hz, 1H), 8.74 (s, 1H), 8.24 – 8.19 (m, 1H), 7.60 (d, J = 7.9 Hz, 1H), 7.53 (dd, J = 6.1, 2.4 Hz, 1H), 7.34 (d, J = 8.1 Hz, 1H), 7.29 – 7.22 (m, 2H), 7.19 (d, J = 2.2 Hz, 1H), 7.09 – 7.04 (m, 1H), 7.02 – 6.96 (m, 1H), 3.53 – 3.50 (m, 3H), 2.95 (t, J = 7.5 Hz, 2H).

^{13}C NMR (126 MHz, DMSO-*d*$_6$) δ 182.3, 163.6, 138.6, 136.4, 136.3, 127.4, 126.3, 123.5, 122.8, 122.7, 121.4, 121.1, 118.4, 118.4, 112.7, 112.2, 111.7, 111.5, 39.8, 24.9.

***N*-(2-(1*H*-Indol-3-yl)ethyl)-2-oxo-2-phenylacetamide (10)**

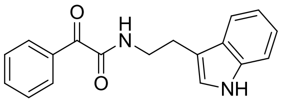

10

Yield: 47% (52.7 mg)

HRMS (*m/z*): calc. for $C_{18}H_{16}N_2O_2$ $[M+H]^+$: 293.1285; found: 293.1284.

^{1}H NMR (500 MHz, Chloroform-*d*) δ 8.31 (dd, *J* = 8.3, 1.2 Hz, 2H), 8.05 (s, 1H), 7.67 – 7.59 (m, 2H), 7.47 (t, *J* = 7.8 Hz, 2H), 7.39 (d, *J* = 8.1 Hz, 1H), 7.24 – 7.20 (m, 1H), 7.18 – 7.12 (m, 2H), 7.11 – 7.08 (m, 1H), 3.75 (q, *J* = 6.8 Hz, 2H), 3.09 (t, *J* = 6.8 Hz, 2H).

^{13}C NMR (126 MHz, Chloroform-*d*) δ 188.0, 161.9, 136.6, 134.5, 133.5, 131.3, 128.6, 127.3, 122.5, 122.3, 119.8, 118.8, 112.7, 111.4, 39.7, 25.3.

(*E*)-*N*-(2-(1*H*-Indol-3-yl)ethyl)-3-methylpent-2-enamide (11)

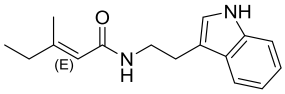

11

Yield: 59% (44.2 mg)

HRMS (*m/z*): calc. for $C_{16}H_{20}N_2O$ $[M+H]^+$: 257.1648; found: 257.1652.

^{1}H NMR (500 MHz, Chloroform-*d*) δ 8.55 (s, 1H), 7.61 (d, *J* = 7.9 Hz, 1H), 7.37 (d, *J* = 8.1 Hz, 1H), 7.24 – 7.15 (m, 1H), 7.15 – 7.08 (m, 1H), 7.00 (d, *J* = 2.2 Hz, 1H), 5.60 (s, 1H), 5.49 – 5.42 (m, 1H), 3.65 (q, *J* = 6.7 Hz, 2H), 2.99 (t, *J* = 6.8 Hz, 2H), 2.15 (d, *J* = 1.0 Hz, 3H), 2.08 (q, *J* = 7.2 Hz, 2H), 1.03 (t, *J* = 7.5 Hz, 3H).

^{13}C NMR (126 MHz, Chloroform-*d*) δ 167.5, 155.8, 136.6, 127.4, 122.3, 122.1, 119.4, 118.8, 117.1, 113.0, 111.4, 39.6, 33.5, 25.5, 18.3, 12.2.

(*Z*)-*N*-(2-(1*H*-Indol-3-yl)ethyl)-3-methylpent-2-enamide (12)

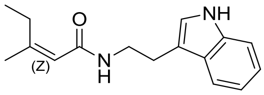

12

Yield: 59% (15.8 mg)

HRMS (*m/z*): calc. for $C_{16}H_{20}N_2O$ $[M+H]^+$: 257.1648; found: 257.1650.

^{1}H NMR (500 MHz, Chloroform-*d*) δ 8.28 (s, 1H), 7.61 (d, *J* = 7.9 Hz, 1H), 7.37 (d, *J* = 8.1 Hz, 1H), 7.23 – 7.17 (m, 1H), 7.15 – 7.09 (m, 1H), 7.03 (d, *J* = 2.2 Hz, 1H), 5.47 (s, 1H), 5.42 (s, 1H), 3.64 (q, *J* = 6.7 Hz, 2H), 2.99 (t, *J* = 6.7 Hz, 2H), 2.64 (q, *J* = 7.6 Hz, 2H), 1.79 (d, *J* = 1.1 Hz, 3H), 1.06 (t, *J* = 7.6 Hz, 3H).

^{13}C NMR (126 MHz, Chloroform-*d*) δ 166.9, 156.2, 136.5, 127.5, 122.2 (two overlapping signals), 119.5, 118.9, 118.2, 113.2, 111.4, 39.5, 26.2, 25.5, 24.3, 12.9.

2-Chloro-1-(1*H*-pyrrolo[3,2-b]pyridin-3-yl)ethanone (14)

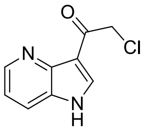

14

Yield: 67% (1.35 g)

^{1}H NMR (400 MHz, DMSO-*d*$_6$) δ 12.49 (s, 1H), 8.54 (dd, *J* = 4.7, 1.4 Hz, 1H), 8.47 (s, 1H), 7.96 (dd, *J* = 8.2, 1.5 Hz, 1H), 7.29 (dd, *J* = 8.2, 4.7 Hz, 1H), 5.24 (s, 2H).

3-(2-Chloroethyl)-1*H*-pyrrolo[3,2-b]pyridine (15)

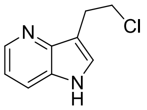

15

Yield: 93% (1.14 g)

^{1}H NMR (250 MHz, DMSO-*d*$_6$) δ 11.12 (s, 1H), 8.30 (dd, *J* = 4.6, 1.4 Hz, 1H), 7.73 (dd, *J* = 8.2, 1.4 Hz, 1H), 7.53 (d, *J* = 2.7 Hz, 1H), 7.09 (dd, *J* = 8.2, 4.6 Hz, 1H), 3.94 (t, *J* = 7.4 Hz, 2H), 3.26 – 3.16 (m, 2H).

2-(2-(1*H*-Pyrrolo[3,2-*b*]pyridin-3-yl)ethyl)isoindoline-1,3-dione (16)

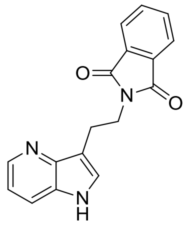

16

Yield: 21% (346 mg)

^{1}H NMR (500 MHz, DMSO-*d*$_6$) δ 11.01 (s, 1H), 8.06 (dd, *J* = 4.5, 1.4 Hz, 1H), 7.80 (s, 4H), 7.65 (dd, *J* = 8.1, 1.4 Hz, 1H), 7.45 (d, *J* = 2.6 Hz, 1H), 6.99 (dd, *J* = 8.1, 4.5 Hz, 1H), 3.92 (t, *J* = 7.1 Hz, 2H), 3.09 (t, *J* = 7.1 Hz, 2H).

^{13}C NMR (126 MHz, DMSO-*d*$_6$) δ 167.9, 145.1, 141.5, 134.2, 131.8, 128.5, 126.7, 122.8, 118.2, 116.1, 111.5, 38.4, 22.9.

2-(2-(1-Methyl-1*H*-pyrrolo[3,2-*b*]pyridin-3-yl)-2-ethyl)isoindoline-1,3-dione (17)

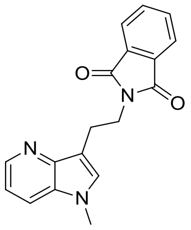

17

Yield: 62% (95.0 mg)

^{1}H NMR (500 MHz, Chloroform-*d*) δ 8.27 (dd, *J* = 4.6, 1.3 Hz, 1H), 7.78 (dd, *J* = 5.4, 3.0 Hz, 2H), 7.67 (dd, *J* = 5.4, 3.1 Hz, 2H), 7.53 (dd, *J* = 8.2, 1.4 Hz, 1H), 7.12 (s, 1H), 7.04 (dd, *J* = 8.2, 4.6 Hz, 1H), 4.08 (t, *J* = 7.0 Hz, 2H), 3.72 (s, 3H), 3.28 (t, *J* = 7.0 Hz, 2H).

^{13}C NMR (126 MHz, Chloroform-*d*) δ 168.5, 145.5, 142.3, 133.8, 132.5, 130.1, 130.1 123.2, 116.4, 116.4, 112.3, 38.7, 32.9, 23.3.

2-(1*H*-Pyrrolo[3,2-*b*]pyridin-3-yl)ethylamine (18)

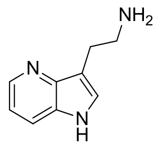

4ATRA (**18**)

Yield: 99% (62.2 mg)

^{1}H NMR (400 MHz, Chloroform-*d*) δ 9.78 (s, 1H), 8.43 (dd, *J* = 4.7, 1.4 Hz, 1H), 7.60 (dd, *J* = 8.2, 1.4 Hz, 1H), 7.25 (s, 1H), 7.06 (dd, *J* = 8.2, 4.6 Hz, 1H), 3.08 (td, *J* = 6.3, 1.3 Hz, 2H), 3.00 (dd, *J* = 7.2, 5.1 Hz, 2H), 1.77 (s, 2H).

2-(1-Methyl-1*H*-pyrrolo[3,2-*b*]pyridin-3-yl)ethylamine (19)

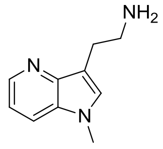

1M4ATRA (**19**)

Yield: quantitative (14.5 mg)

^{1}H NMR (500 MHz, Chloroform-*d*) δ 8.47 – 8.40 (m, 1H), 7.57 (dd, *J* = 8.2, 1.4 Hz, 1H), 7.14 – 7.06 (m, 2H), 3.76 (s, 3H), 3.06 (td, *J* = 6.4, 1.3 Hz, 2H), 2.99 (t, *J* = 6.4 Hz, 2H), 2.55 – 2.30 (m, 2H).

^{13}C NMR (126 MHz, Chloroform-*d*) δ 145.7, 142.4, 130.2 (two overlapping signals), 116.4, 116.4, 113.5, 42.8, 32.9, 28.4.

2-Chloro-1-(1*H*-pyrrolo[2,3-b]pyridin-3-yl)ethanone (21)

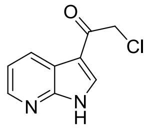

21

Yield: 32% (585 mg)

^{1}H NMR (400 MHz, DMSO-d_6) δ 12.66 (s, 1H), 8.58 (d, *J* = 2.7 Hz, 1H), 8.46 (dd, *J* = 7.9, 1.4 Hz, 1H), 8.35 (dd, *J* = 4.7, 1.5 Hz, 1H), 7.28 (dd, *J* = 7.9, 4.7 Hz, 1H), 4.92 (s, 2H).

^{13}C NMR (75 MHz, DMSO-d_6) δ 187.3, 149.6, 145.2, 135.7, 130.0, 118.9, 118.2, 112.8, 46.3.

3-(2-Chloroethyl)-1*H*-pyrrolo[2,3-*b*]pyridine (22)

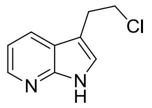

22

Yield: 91% (745 mg)

^{1}H NMR (400 MHz, DMSO-d_6) δ 11.45 (s, 1H), 8.19 (dd, *J* = 4.7, 1.5 Hz, 1H), 8.00 (dd, *J* = 7.9, 1.4 Hz, 1H), 7.36 (d, *J* = 2.2 Hz, 1H), 7.03 (dd, *J* = 7.9, 4.7 Hz, 1H), 3.86 (t, *J* = 7.2 Hz, 2H), 3.18 – 3.10 (m, 2H).

^{13}C NMR (75 MHz, DMSO-d_6) δ 148.5, 142.5, 126.6, 124.1, 119.2, 115.0, 109.7, 45.1, 28.5.

2-(2-(1*H*-Pyrrolo[2,3-*b*]pyridin-3-yl)ethyl)isoindoline-1,3-dione (23)

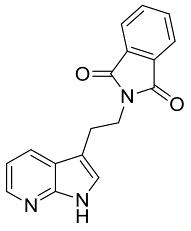

23

Yield: 56% (661 mg)

^{1}H NMR (500 MHz, DMSO-d_6) δ 11.36 (s, 1H), 8.17 (d, *J* = 4.6 Hz, 1H), 7.93 (d, *J* = 7.8 Hz, 1H), 7.86 – 7.77 (m, 4H), 7.28 (d, *J* = 1.9 Hz, 1H), 7.01 (dd, *J* = 7.8, 4.7 Hz, 1H), 3.85 (t, *J* = 7.4 Hz, 2H), 3.03 (t, *J* = 7.3 Hz, 2H).

^{13}C NMR (126 MHz, DMSO-d_6) δ 167.8, 148.6, 142.5, 134.4, 131.6, 126.2, 123.6, 123.0, 119.3, 114.9, 109.7, 38.1, 23.9.

2-(2-(1-Methyl-1*H*-pyrrolo[2,3-*b*]pyridin-3-yl)-2-oxoethyl)isoindoline-1,3-dione (24)

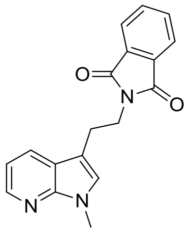

24

Yield: 57% (87.6 mg)

^{1}H NMR (500 MHz, Chloroform-*d*) δ 8.31 (dd, *J* = 4.7, 1.5 Hz, 1H), 7.99 (dd, *J* = 7.8, 1.5 Hz, 1H), 7.82 (dd, *J* = 5.4, 3.0 Hz, 2H), 7.70 (dd, *J* = 5.4, 3.0 Hz, 2H), 7.07 (s, 1H), 7.03 (dd, *J* = 7.8, 4.7 Hz, 1H), 3.99 – 3.92 (m, 2H), 3.83 (s, 3H), 3.15 – 3.07 (m, 2H).

^{13}C NMR (126 MHz, Chloroform-*d*) δ 168.4, 148.0, 143.0, 134.1, 132.2, 127.2, 127.0, 123.3, 120.3, 115.2, 109.5, 38.6, 31.2, 24.6.

2-(1*H*-Pyrrolo[2,3-*b*]pyridin-3-yl)ethylamine (25)

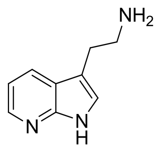

7ATRA (**25**)

Yield: 92% (74.3 mg)

^{1}H NMR (500 MHz, Chloroform-*d*) δ 11.57 (s, 1H), 8.29 (dd, *J* = 4.8, 1.6 Hz, 1H), 7.91 (dd, *J* = 7.8, 1.6 Hz, 1H), 7.17 (s, 1H), 7.04 (dd, *J* = 7.8, 4.8 Hz, 1H), 3.01 (t, *J* = 6.7 Hz, 2H), 2.87 (t, *J* = 6.7 Hz, 2H), 1.66 (s, 2H).

^{13}C NMR (126 MHz, Chloroform-*d*) δ 149.3, 142.4, 127.4, 123.1, 120.4, 115.1, 111.9, 42.4, 29.6.

2-(1-Methyl-1*H*-pyrrolo[2,3-*b*]pyridin-3-yl)ethylamine (26)

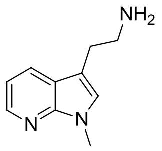

1M7ATRA (**26**)

Yield: quantitative (49.6 mg)

^{1}H NMR (500 MHz, Chloroform-*d*) δ 8.31 (dd, *J* = 4.7, 1.5 Hz, 1H), 7.87 (dd, *J* = 7.8, 1.6 Hz, 1H), 7.05 – 6.97 (m, 2H), 3.84 (s, 3H), 2.99 (t, *J* = 6.7 Hz, 2H), 2.90 – 2.82 (m, 2H), 1.73 (s, 2H).

^{13}C NMR (126 MHz, Chloroform-*d*) δ 148.2, 143.0, 127.2, 126.9, 120.4, 115.0, 110.9, 42.6, 31.1, 29.5.

N-(2-(1H-Pyrrolo[3,2-b]pyridin-3-yl)ethyl)-3-methyl-2-oxopentanamide (27)

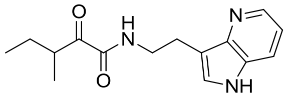

27

Yield: 32% (1.5 mg)

HRMS (*m/z*): calc. for $C_{15}H_{19}N_3O_2$ $[M+H]^+$: 274.1550; found: 274.1549.

^{1}H NMR (500 MHz, Chloroform-*d*) δ 8.71 (s, 1H), 8.53 (dd, *J* = 4.7, 1.4 Hz, 2H), 7.73 (dd, *J* = 8.3, 1.4 Hz, 1H), 7.32 (d, *J* = 2.4 Hz, 1H), 7.17 (dd, *J* = 8.2, 4.7 Hz, 1H), 3.71 – 3.63 (m, 2H), 3.51 – 3.42 (m, 1H), 3.17 – 3.10 (m, 2H), 1.71 (dqd, *J* = 13.7, 7.4, 6.2 Hz, 1H), 1.38 (dt, *J* = 13.6, 7.2 Hz, 1H), 1.07 (d, *J* = 6.9 Hz, 3H), 0.87 (t, *J* = 7.5 Hz, 3H).

^{13}C NMR (126 MHz, Chloroform-*d*) δ 202.5, 160.9, 144.5, 142.3, 129.6, 126.5, 119.4, 117.3, 114.2, 40.7, 40.6, 25.6, 24.4, 15.3, 11.7.

3-Methyl-N-(2-(1-methyl-1H-pyrrolo[3,2-b]pyridin-3-yl)ethyl)-2-oxopentanamide (28)

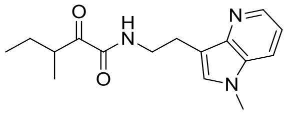

28

Yield: 16% (1.9 mg)

HRMS (*m/z*): calc. for $C_{16}H_{21}N_3O_2$ $[M+H]^+$: 288.1707; found: 288.1704.

^{1}H NMR (500 MHz, Chloroform-*d*) δ 8.52 (d, *J* = 4.8 Hz, 1H), 7.68 (d, *J* = 8.4 Hz, 1H), 7.22 – 7.16 (m, 2H), 3.80 (s, 3H), 3.66 (q, *J* = 6.0 Hz, 2H), 3.46 (q, *J* = 6.8 Hz, 1H), 3.14 (t, *J* = 6.1 Hz, 2H), 1.70 (dd, *J* = 14.1, 6.9 Hz, 1H), 1.40 – 1.35 (m, 1H), 1.07 (d, *J* = 7.0 Hz, 3H), 0.87 (t, *J* = 7.4 Hz, 3H).

^{13}C NMR (126 MHz, Chloroform-*d*) δ 202.5, 160.9, 146.7, 143.7, 130.9, 125.2, 117.7, 116.7, 112.8, 40.7, 33.1, 29.8, 25.5, 24.3, 15.3, 11.7.

***N*-(2-(1*H*-Pyrrolo[2,3-*b*]pyridin-3-yl)ethyl)-3-methyl-2-oxopentanamide (29)**

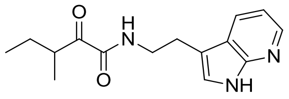

29

Yield: 72% (6.1 mg)

HRMS (*m/z*): calc. for $C_{15}H_{19}N_3O_2$ $[M+H]^+$: 274.1550; found: 274.1553.

^{1}H NMR (500 MHz, Chloroform-*d*) δ 9.53 (s, 1H), 8.31 (dd, *J* = 5.0, 1.5 Hz, 1H), 7.98 (dd, *J* = 7.9, 1.5 Hz, 1H), 7.18 (s, 1H), 7.12 (dd, *J* = 7.8, 4.9 Hz, 2H), 3.63 (td, *J* = 7.0, 6.1 Hz, 2H), 3.53 – 3.43 (m, 1H), 3.01 (t, *J* = 6.8 Hz, 2H), 1.76 – 1.66 (m, 1H), 1.44 – 1.34 (m, 1H), 1.08 (d, *J* = 7.0 Hz, 3H), 0.88 (t, *J* = 7.5 Hz, 3H).

^{13}C NMR (126 MHz, Chloroform-*d*) δ 202.4, 160.2, 148.2, 142.5, 128.0, 122.9, 120.4, 115.8, 111.5, 40.5, 39.7, 25.6, 25.5, 15.3, 11.6.

3-Methyl-*N*-(2-(1-methyl-1*H*-pyrrolo[2,3-*b*]pyridin-3-yl)ethyl)-2-oxopentanamide (30)

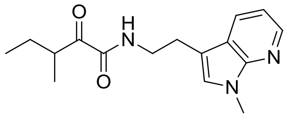

30

Yield: 28% (2.9 mg)

HRMS (*m/z*): calc. for $C_{16}H_{21}N_3O_2$ $[M+H]^+$: 288.1707; found: 288.1705.

^{1}H NMR (500 MHz, Chloroform-*d*) δ 8.35 (dd, *J* = 4.8, 1.5 Hz, 1H), 7.93 (d, *J* = 7.8 Hz, 1H), 7.11 – 7.02 (m, 3H), 3.90 (s, 3H), 3.64 – 3.57 (m, 2H), 3.52 – 3.45 (m, 1H), 2.99 (t, *J* = 6.8 Hz, 2H), 1.76 – 1.66 (m, 1H), 1.44 – 1.34 (m, 1H), 1.08 (d, *J* = 6.9 Hz, 3H), 0.88 (t, *J* = 7.5 Hz, 3H).

^{13}C NMR (126 MHz, Chloroform-*d*) δ 202.4, 160.2, 147.5, 142.3, 136.0, 127.3, 120.5, 115.2, 110.0, 40.5, 39.8, 31.6, 25.6, 25.4, 15.3, 11.6.

Supplementary table

Table S1: MICs of nematophin derivatives in µg/mL against different pathogens. * Median of three experiments.

	**MICs (µg/mL)***			
	***Staphylococcus aureus* (MSSA)**	***Staphylococcus aureus* (MRSA)**	***Micrococcus luteus***	***Enterococcus faecalis***
**compound**	ATCC 29213	ATCC 43300	ATCC 9341	ATCC 29212
**1**	0.5	0.5	32	>128
**2**	16	32	>64	>64
**3**	0.125	0.031	16	>128
**4**	2	4	>64	>64
**5**	1	4	16	>64
**6**	1	1	32	>64
**7**	>128	>128	>128	>128
**8**	64	>64	>64	>64
**9**	>64	>64	>64	>64
**10**	8	8	>64	>64
**11**	>128	>128	>128	>128
**12**	>128	>128	>128	>128
**27**	32	64	128	>128
**28**	16	>128	128	>128
**29**	16	64	>128	>128
**30**	4	32	64	>128

NMR Spectra

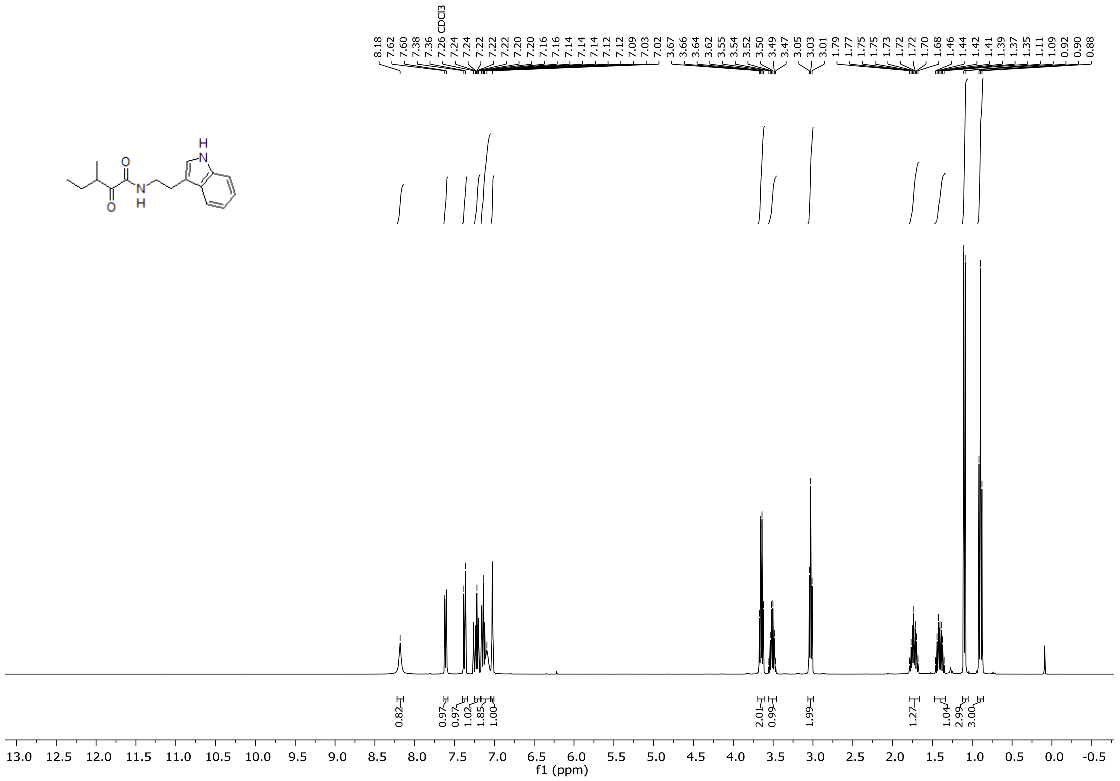

Figure S1: ^{1}H NMR spectra (400 MHz, $CDCl_3$) of **1**.

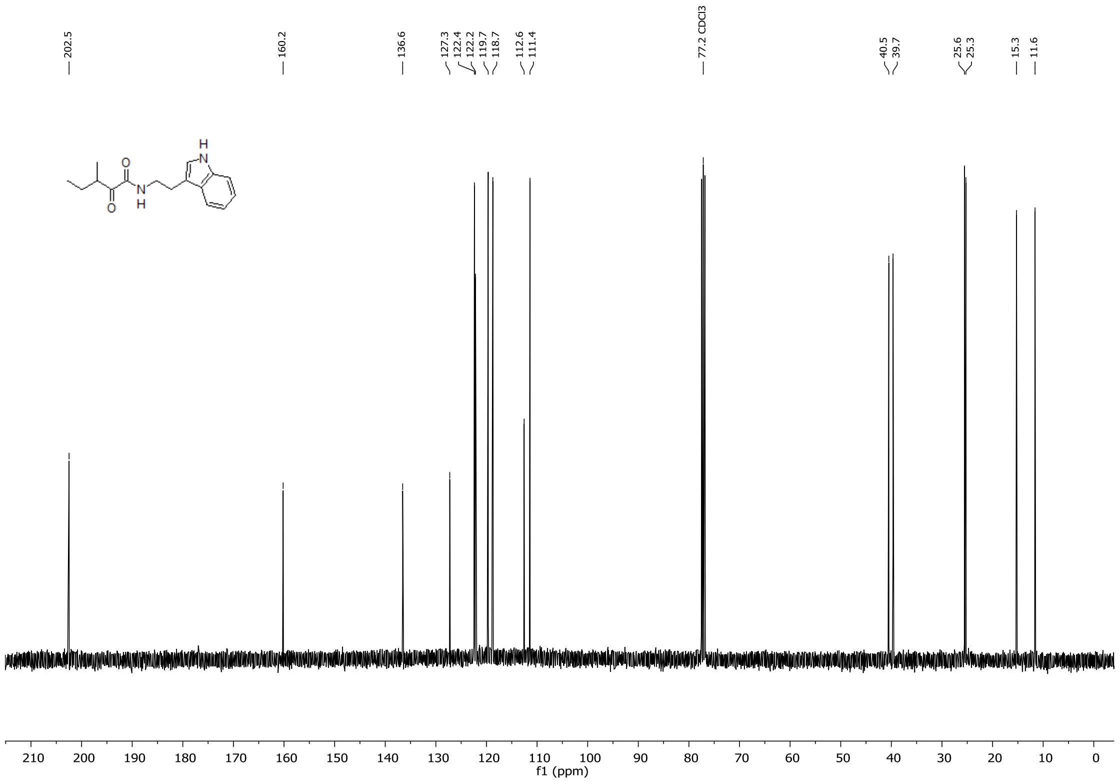

Figure S2: ^{13}C NMR (101 MHz, $CDCl_3$) of **1**.

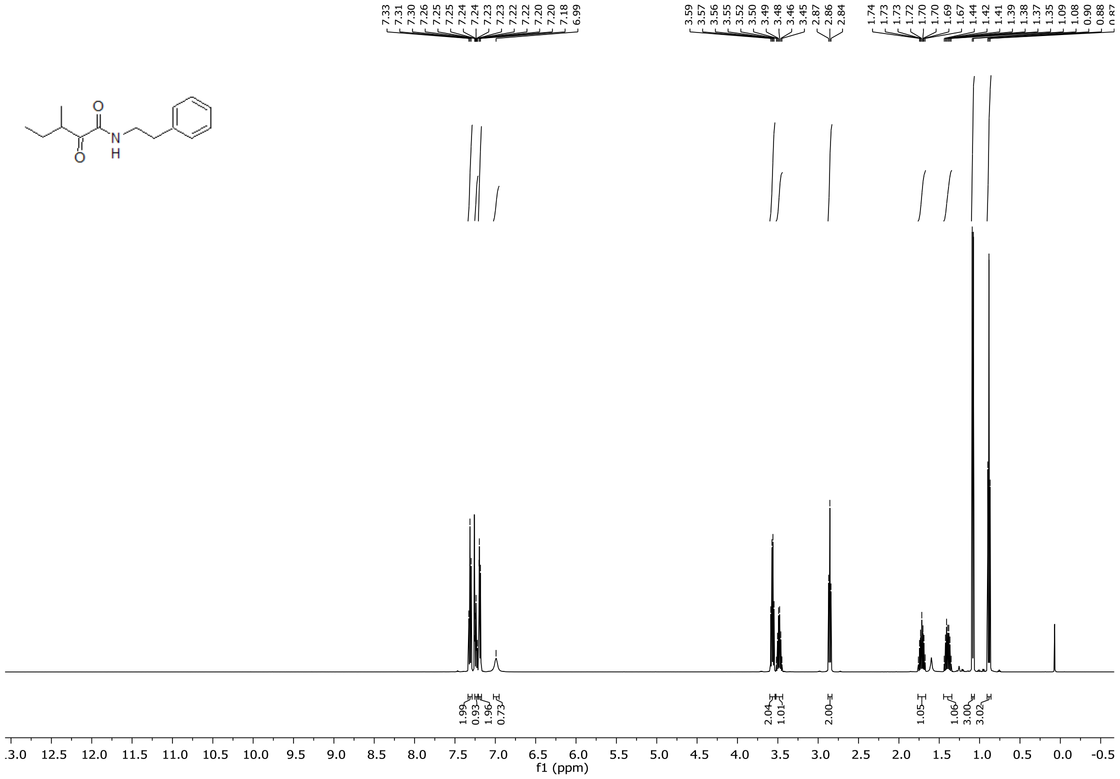

Figure S3: ^{1}H NMR spectra (500 MHz, $CDCl_3$) of **2**.

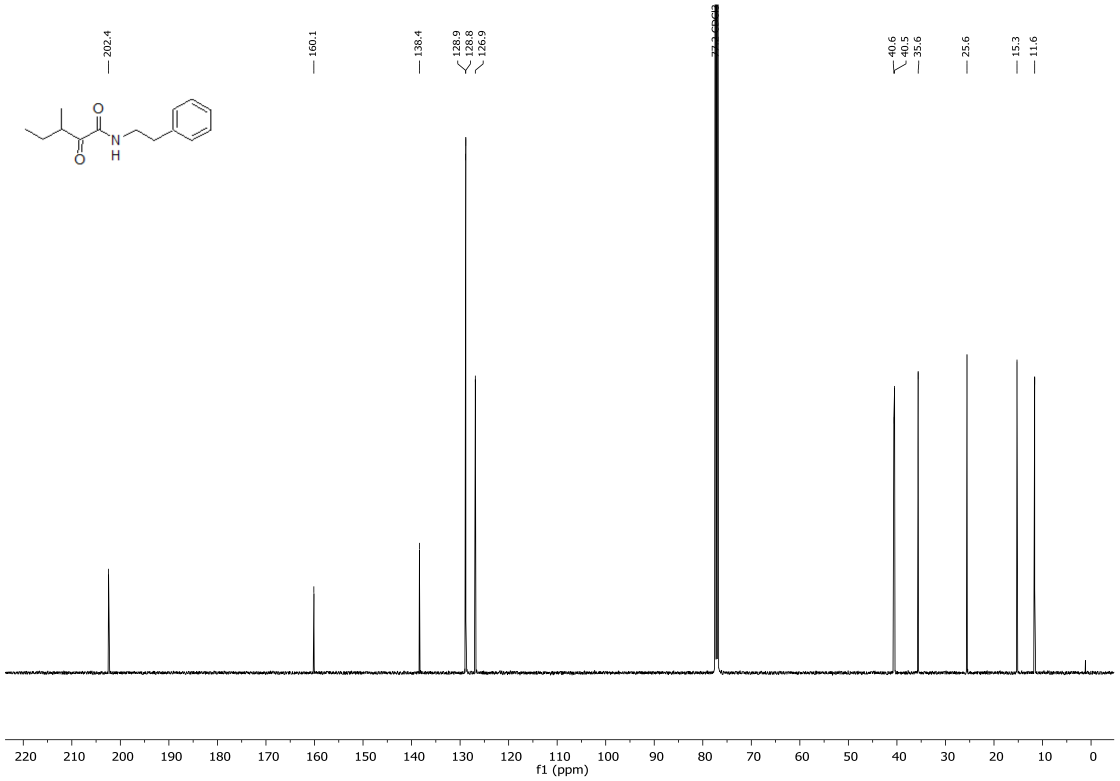

Figure S4: ^{13}C NMR (126 MHz, $CDCl_3$) of **2**.

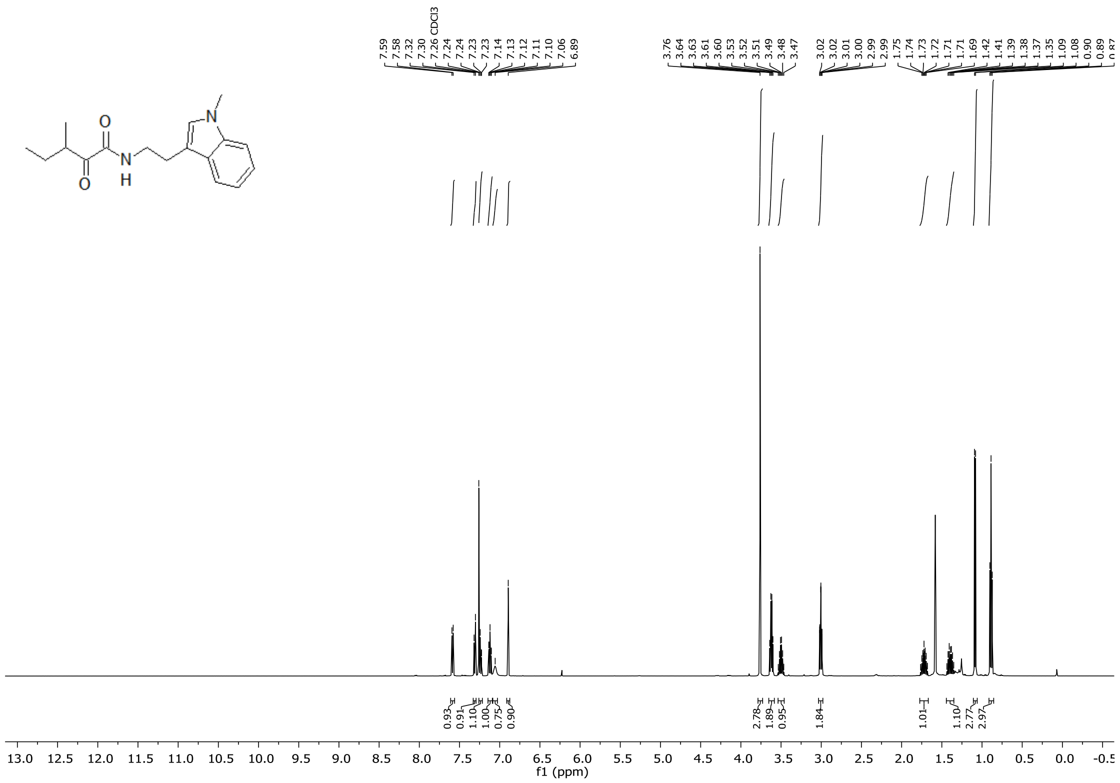

Figure S5: ^{1}H NMR spectra (500 MHz, $CDCl_3$) of **3**.

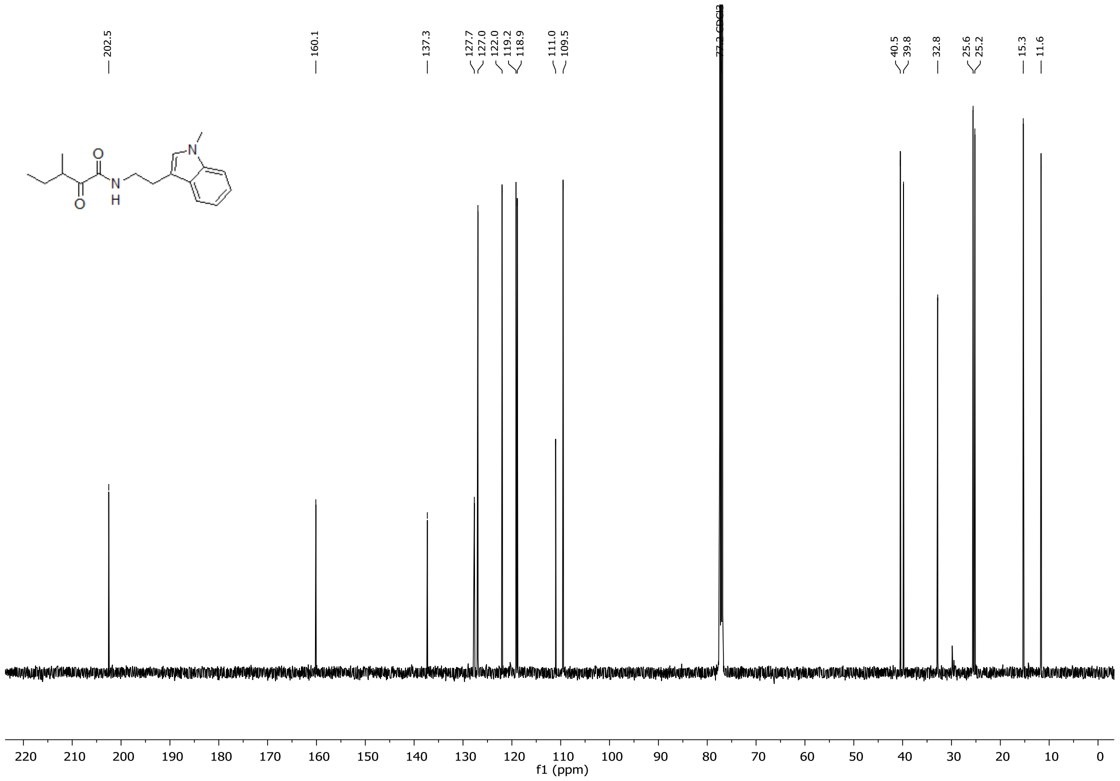

Figure S6: ^{13}C NMR (126 MHz, $CDCl_3$) of **3**.

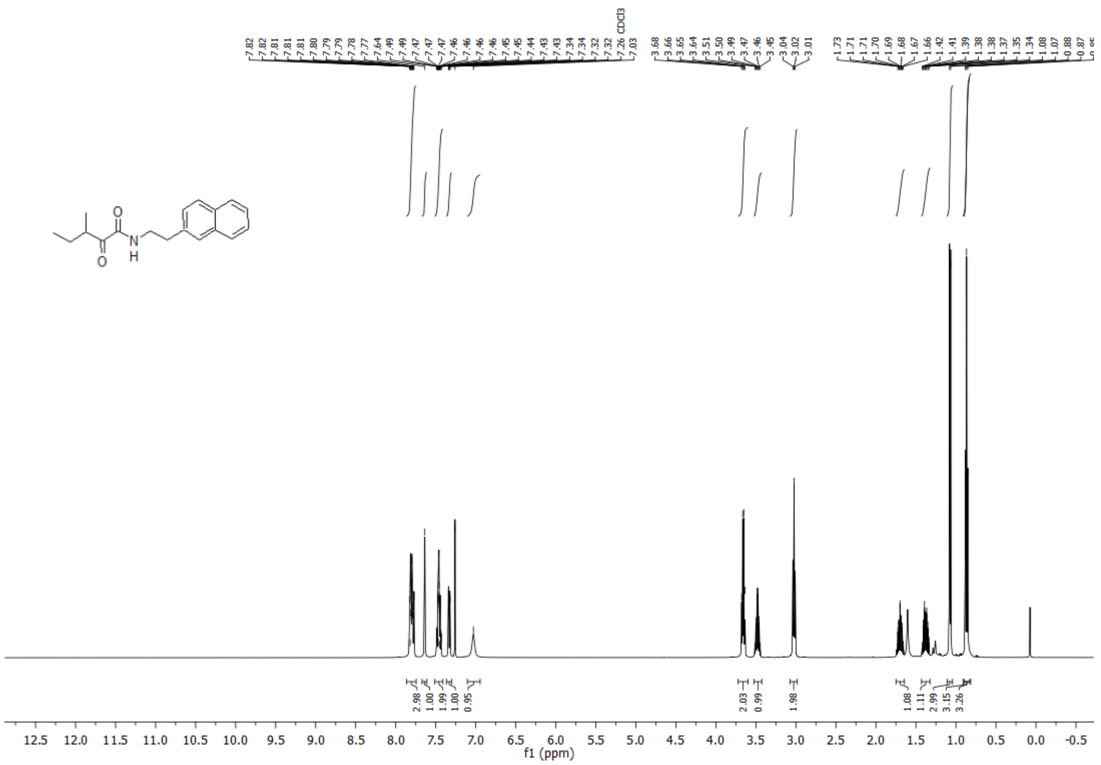

Figure S7: ^{1}H NMR spectra (500 MHz, $CDCl_3$) of **4**.

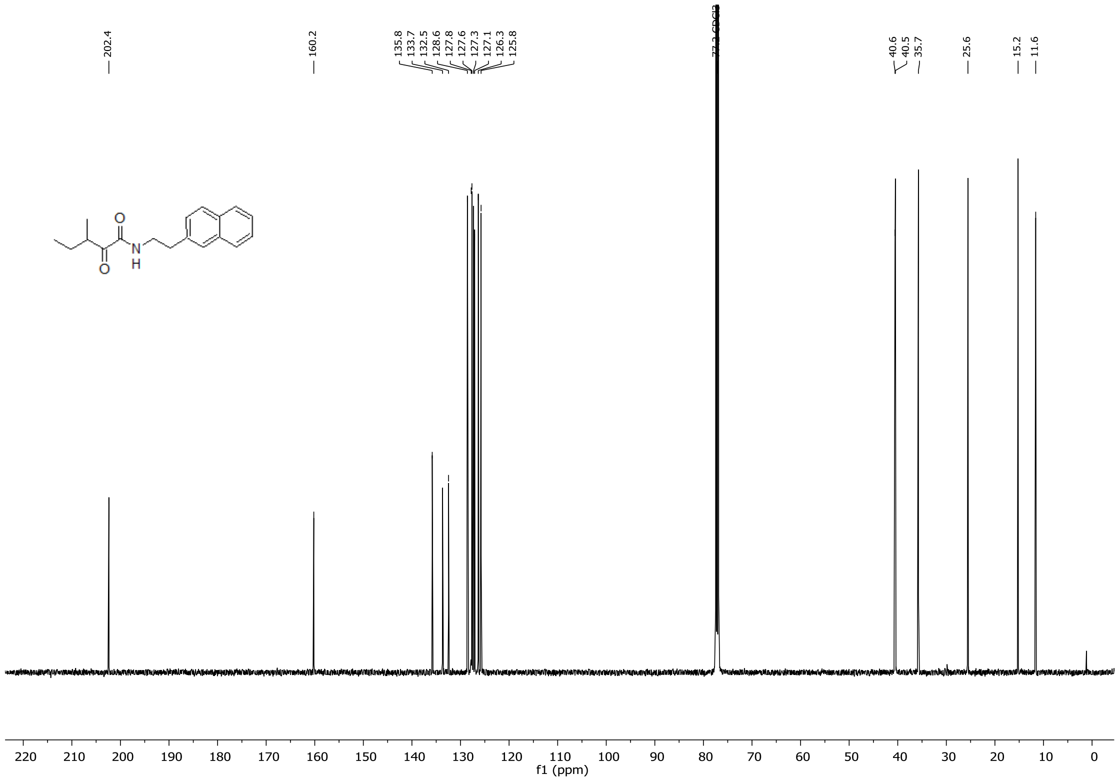

Figure S8: ^{13}C NMR (126 MHz, $CDCl_3$) of **4**.

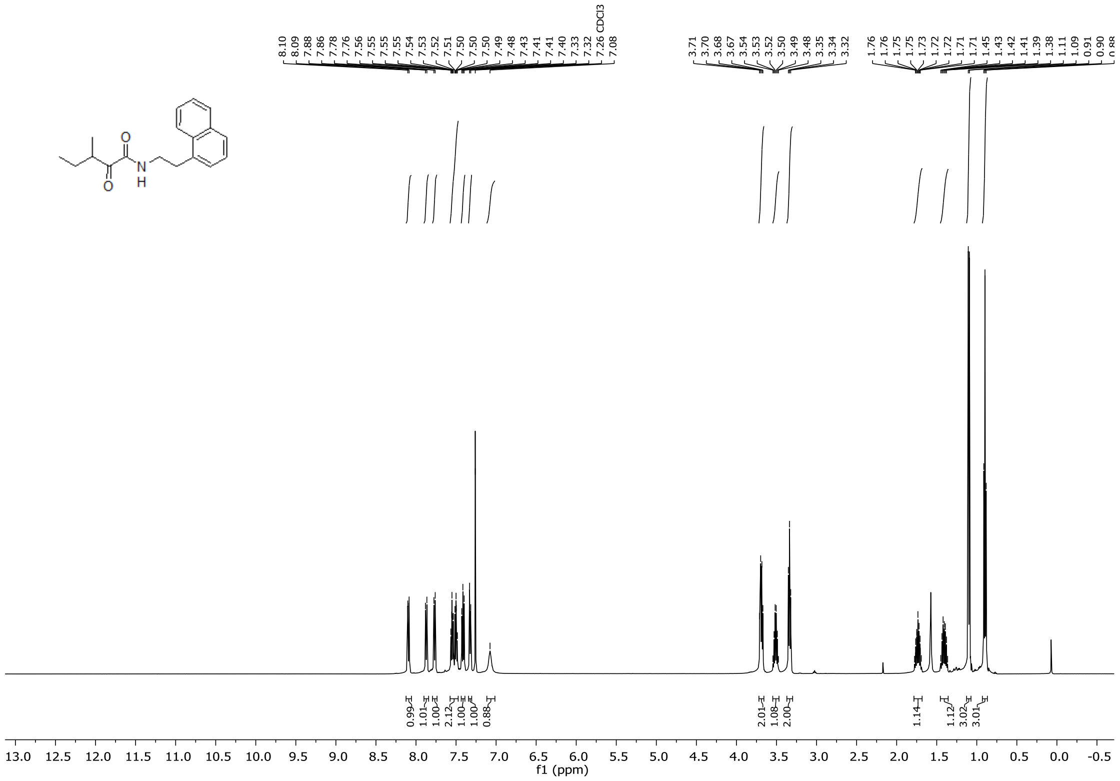

Figure S9: ^{1}H NMR spectra (500 MHz, $CDCl_3$) of **5**.

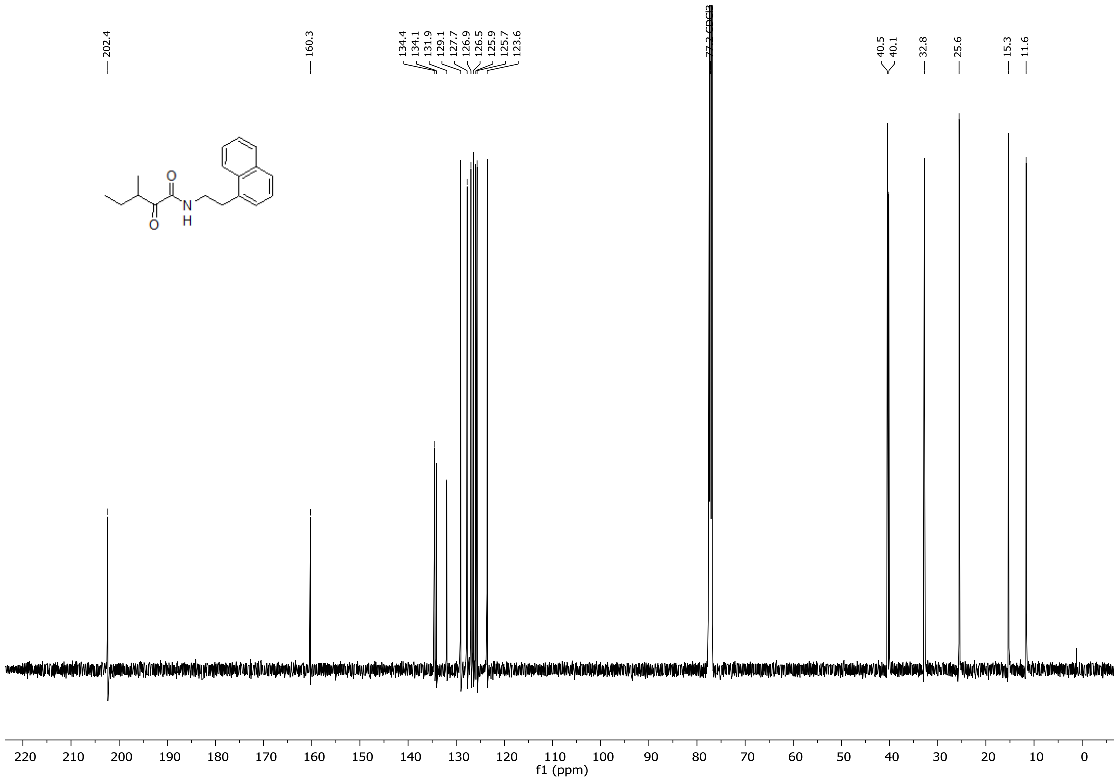

Figure S10: ^{13}C NMR (126 MHz, $CDCl_3$) of **5**.

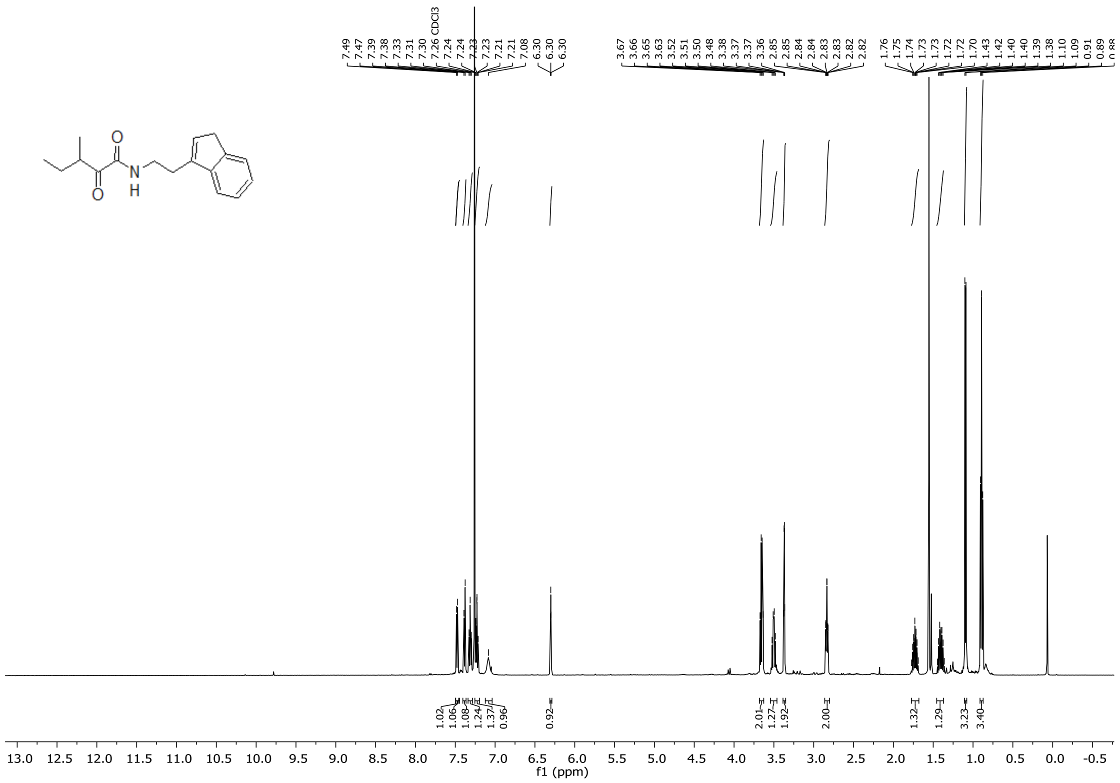

Figure S11: ^{1}H NMR spectra (500 MHz, $CDCl_3$) of **6**.

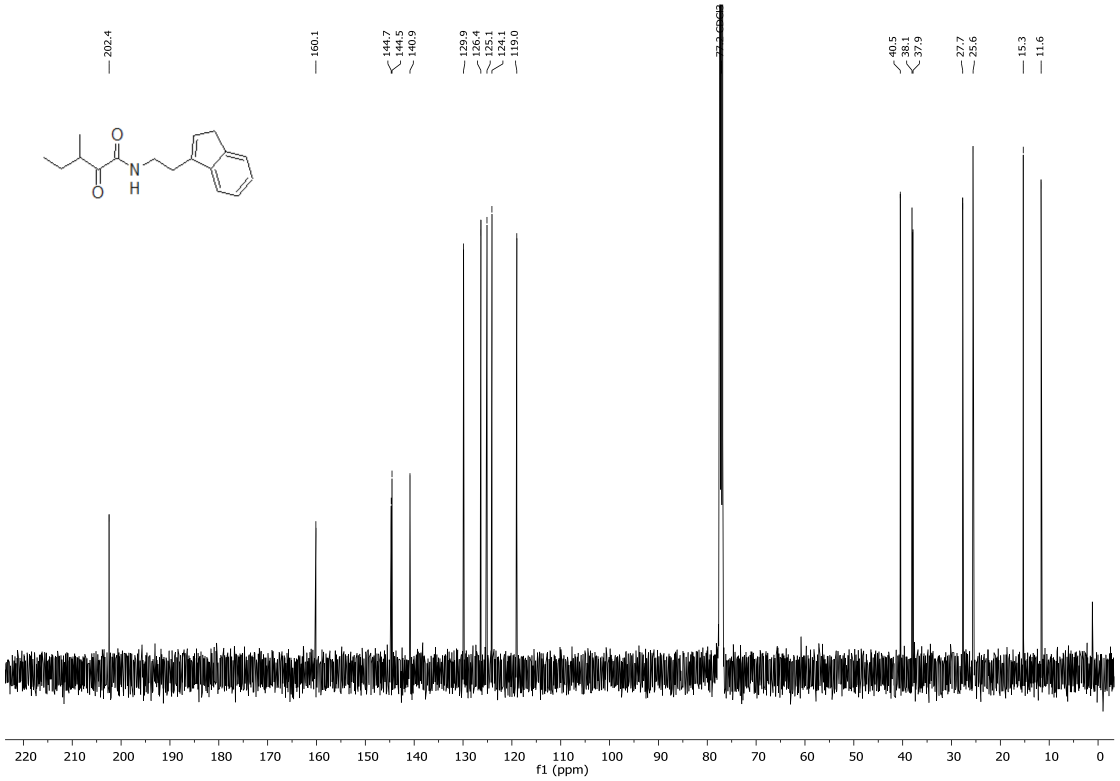

Figure S12: ^{13}C NMR (126 MHz, $CDCl_3$) of **6**.

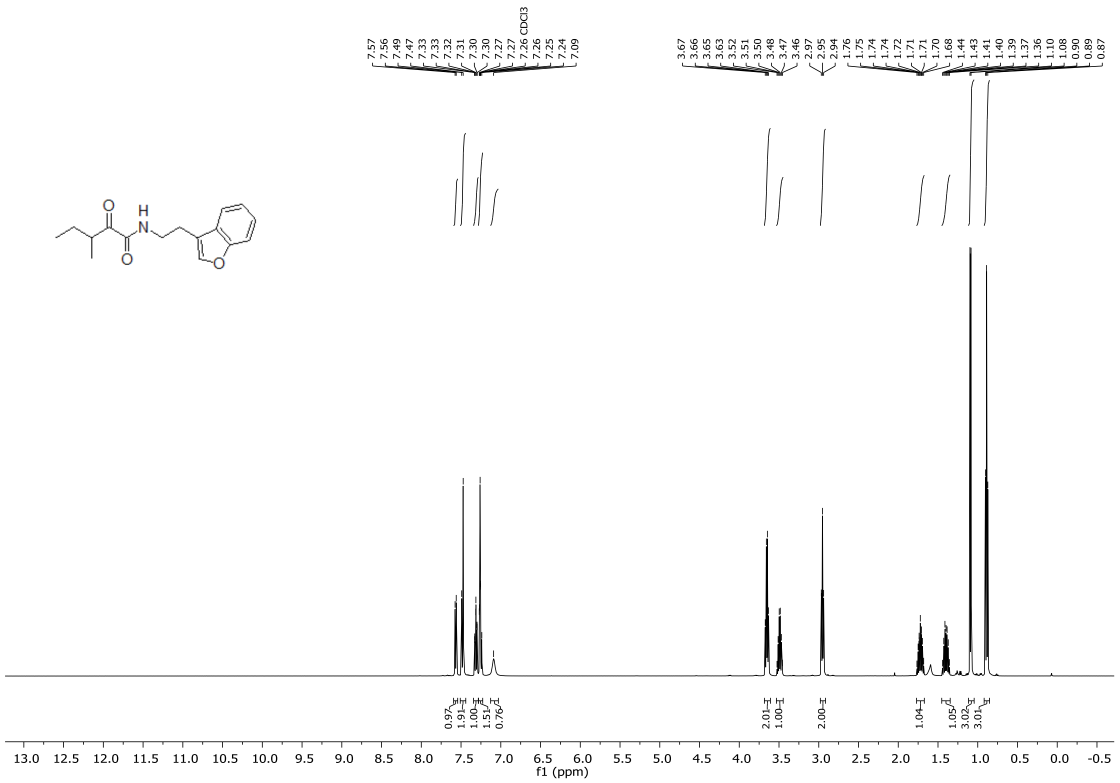

Figure S13: ^{1}H NMR spectra (500 MHz, $CDCl_3$) of **7**.

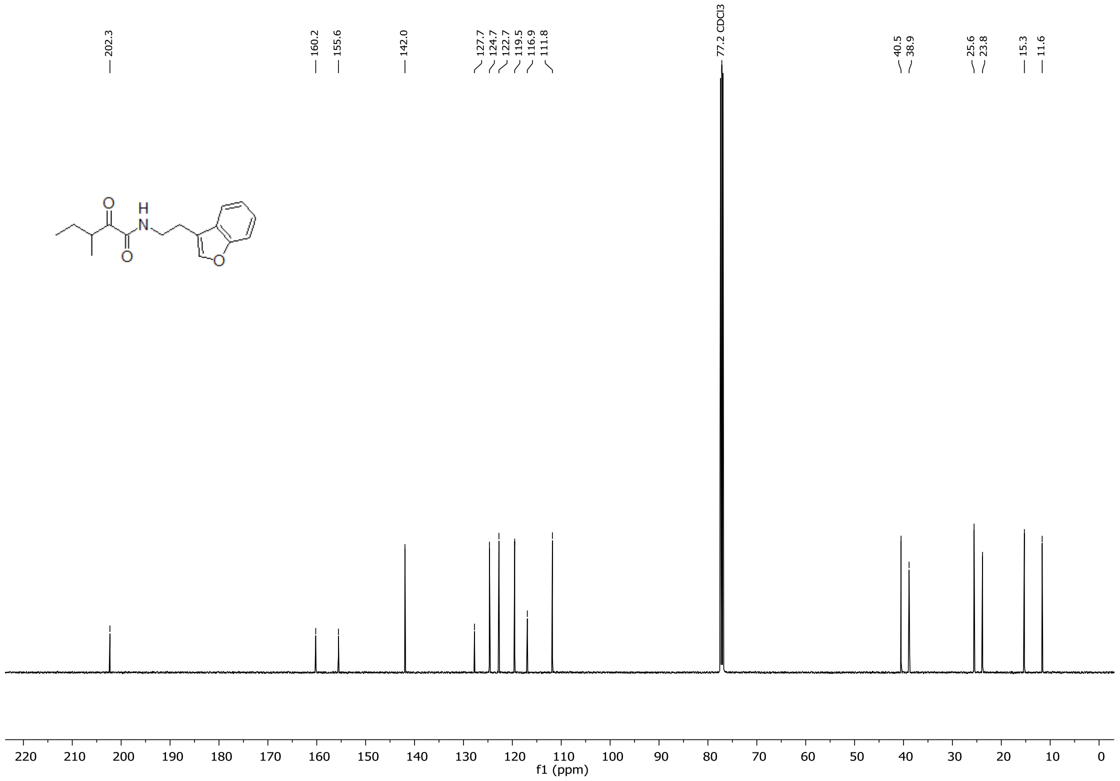

Figure S14: ^{13}C NMR (126 MHz, $CDCl_3$) of **7**.

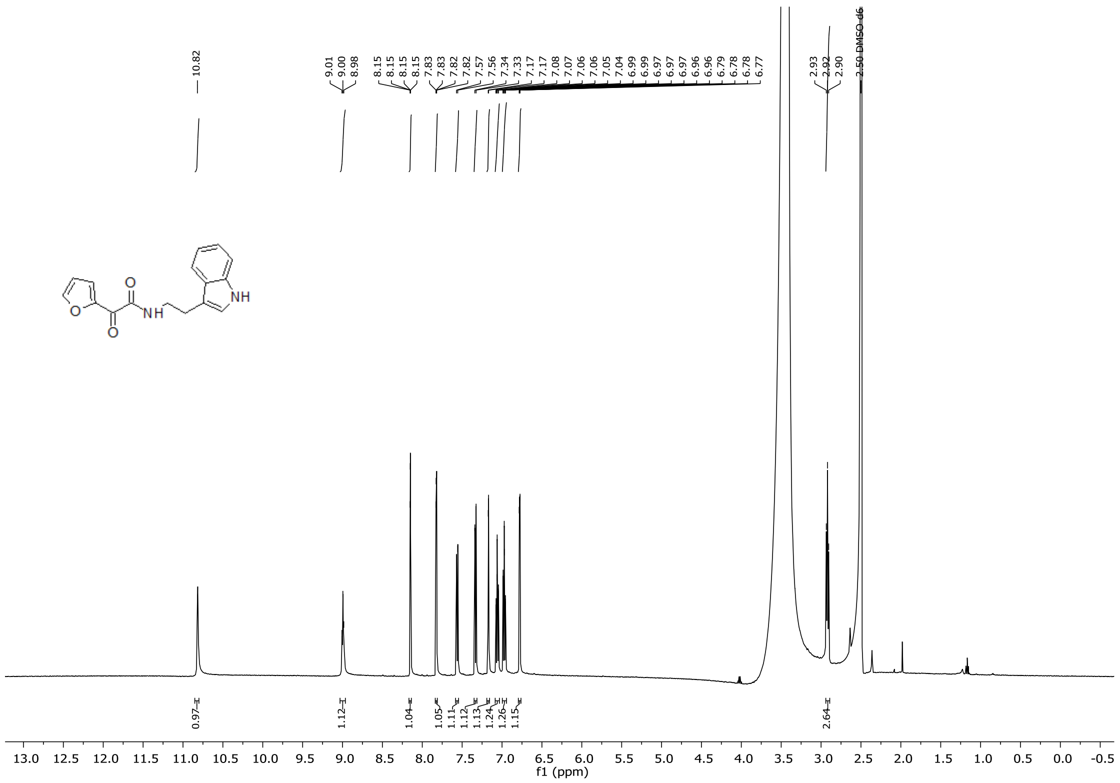

Figure S15: ^{1}H NMR spectra (500 MHz, DMSO-d_6) of **8**.

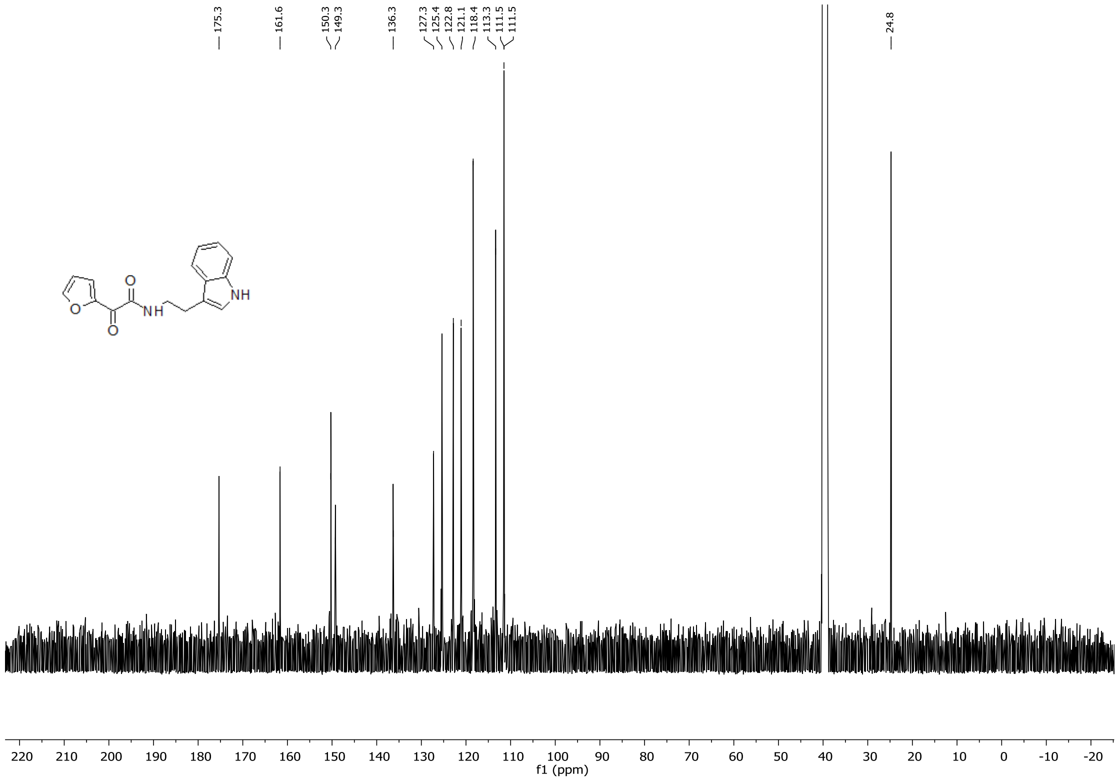

Figure S16: ^{13}C NMR (126 MHz, DMSO-d_6) of **8**.

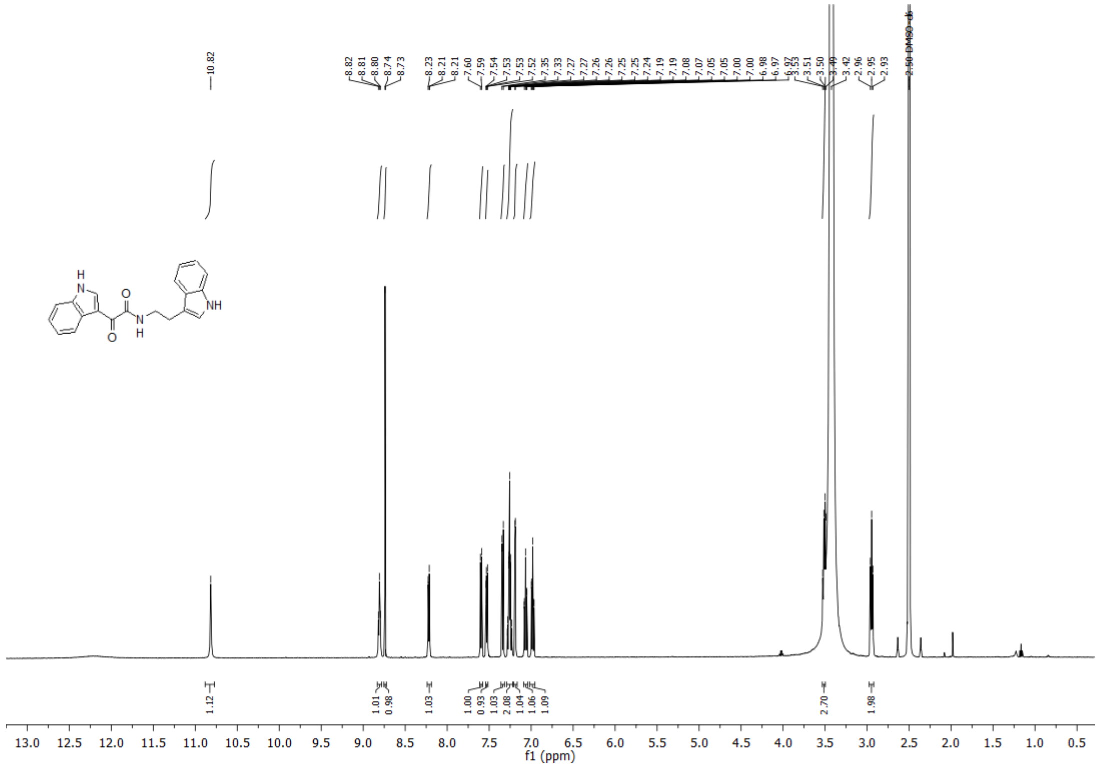

Figure S17: ^{1}H NMR spectra (500 MHz, DMSO-d_6) of **9**.

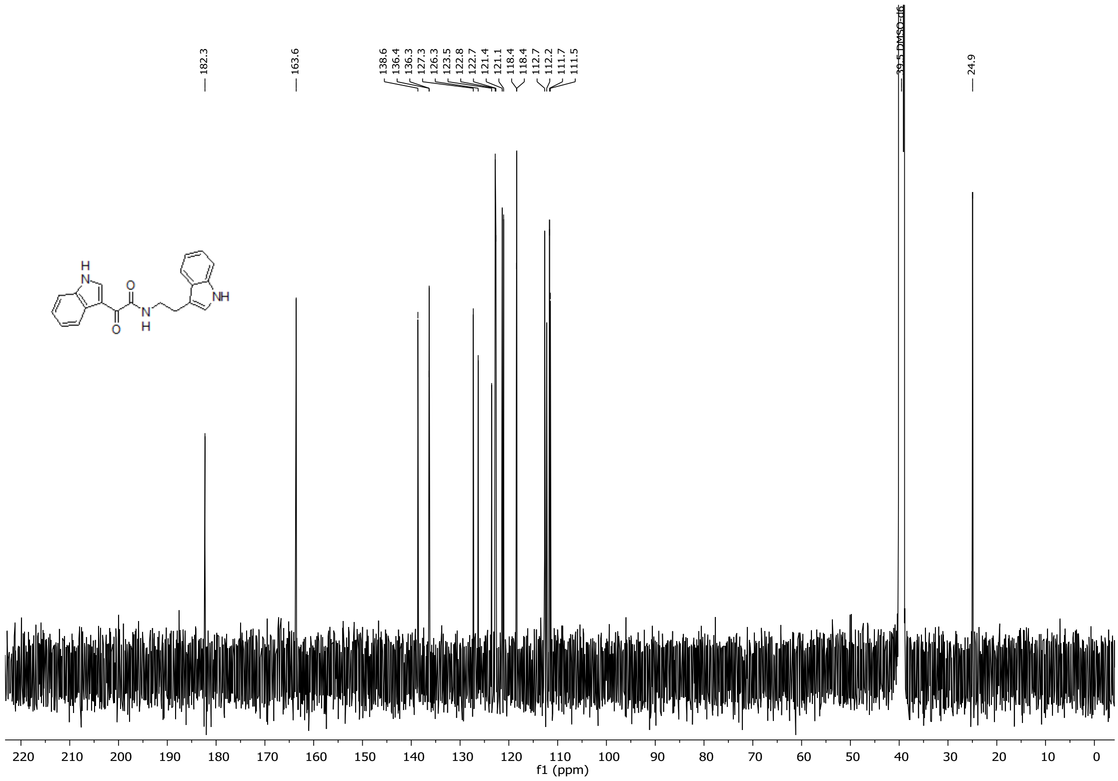

Figure S18: ^{13}C NMR (126 MHz, DMSO-d_6) of **9**.

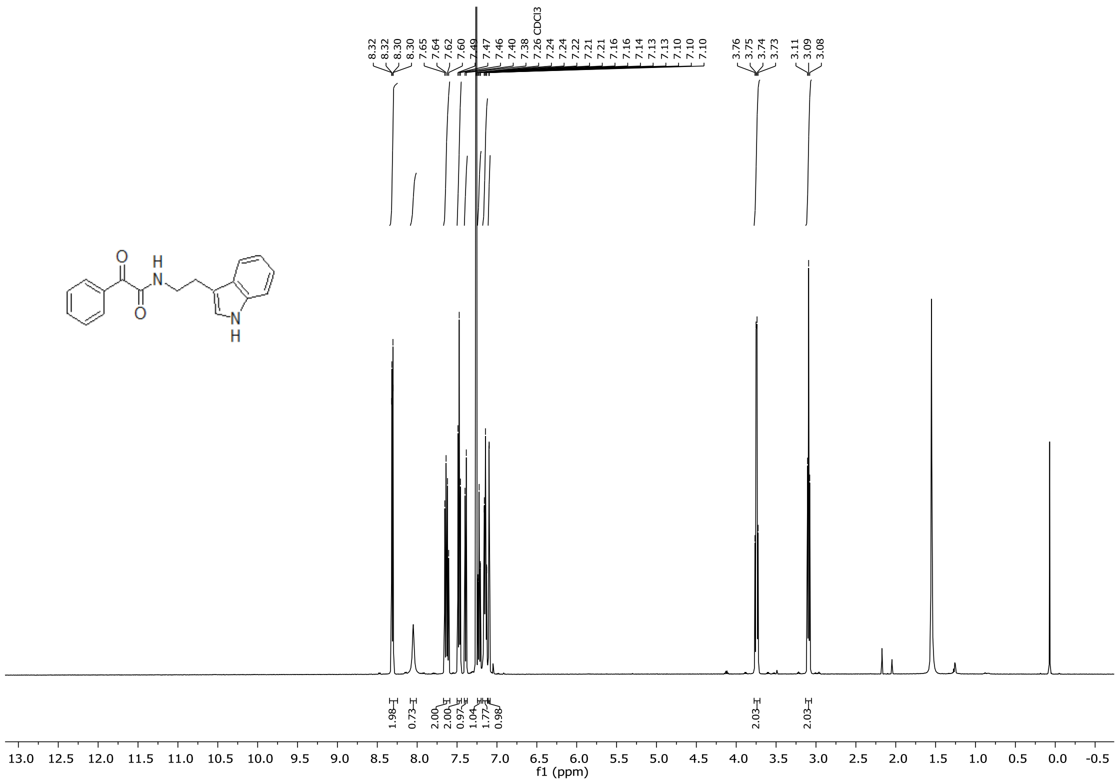

Figure S19: ^{1}H NMR spectra (500 MHz, $CDCl_3$) of **10**.

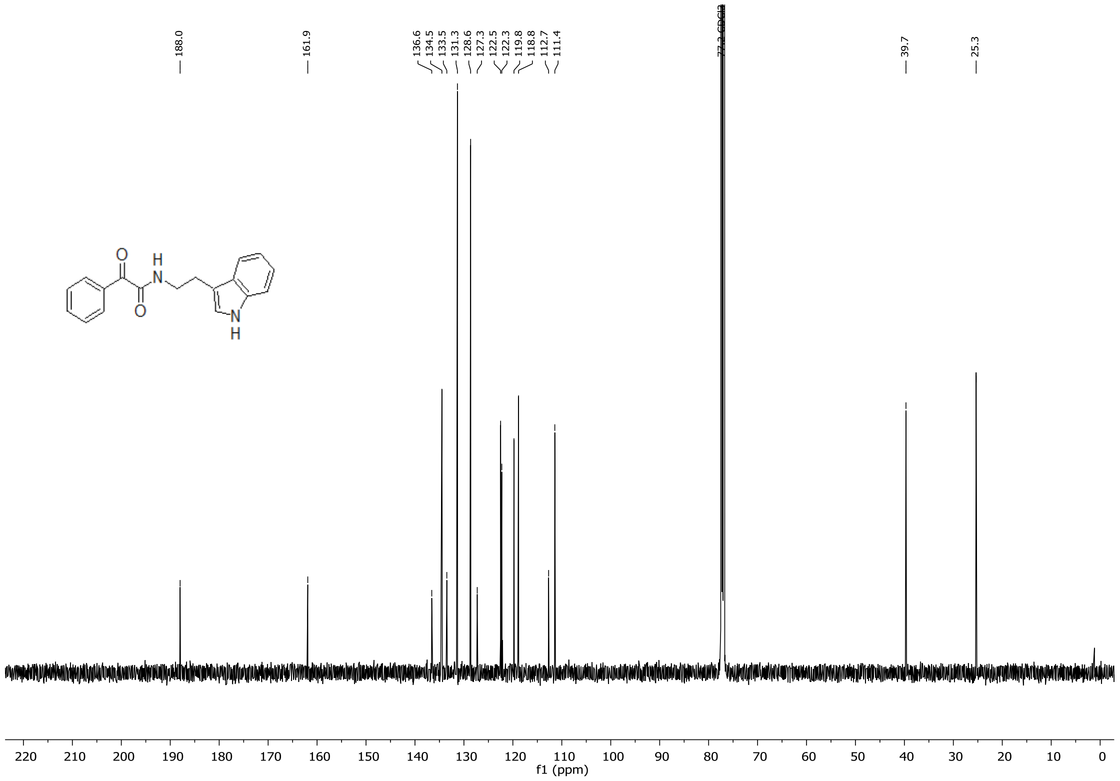

Figure S20: ^{13}C NMR (126 MHz, $CDCl_3$) of **10**.

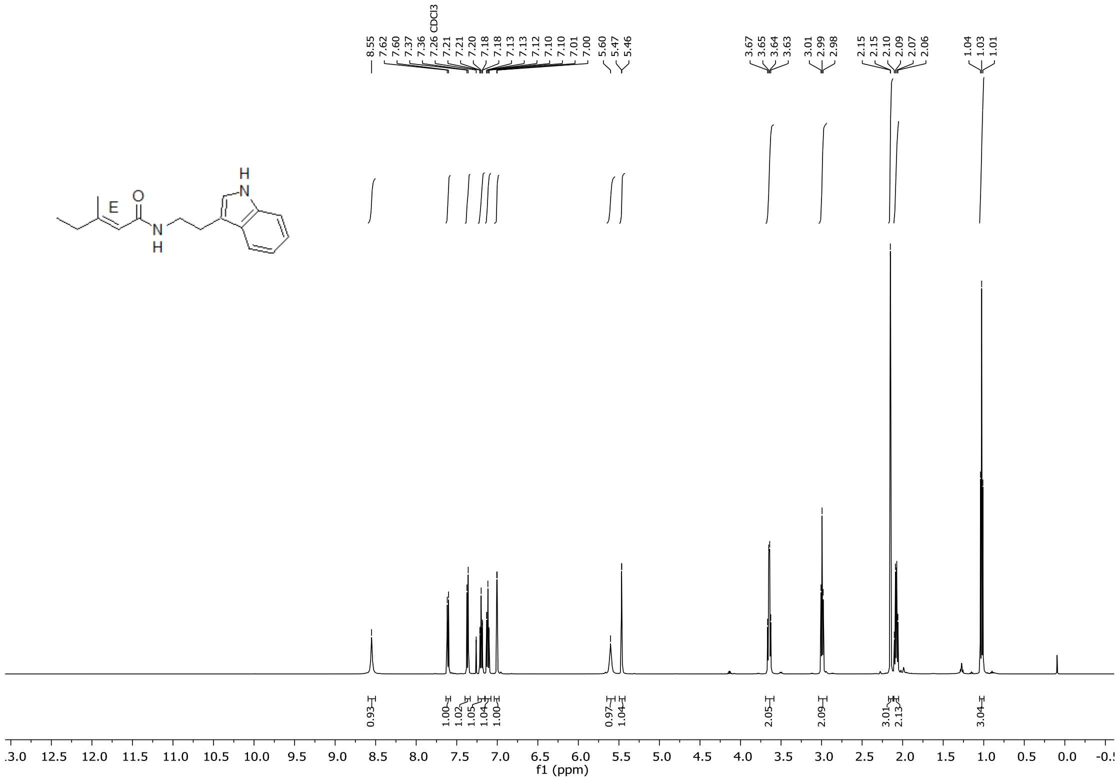

Figure S21: ^{1}H NMR spectra (500 MHz, $CDCl_3$) of **11**.

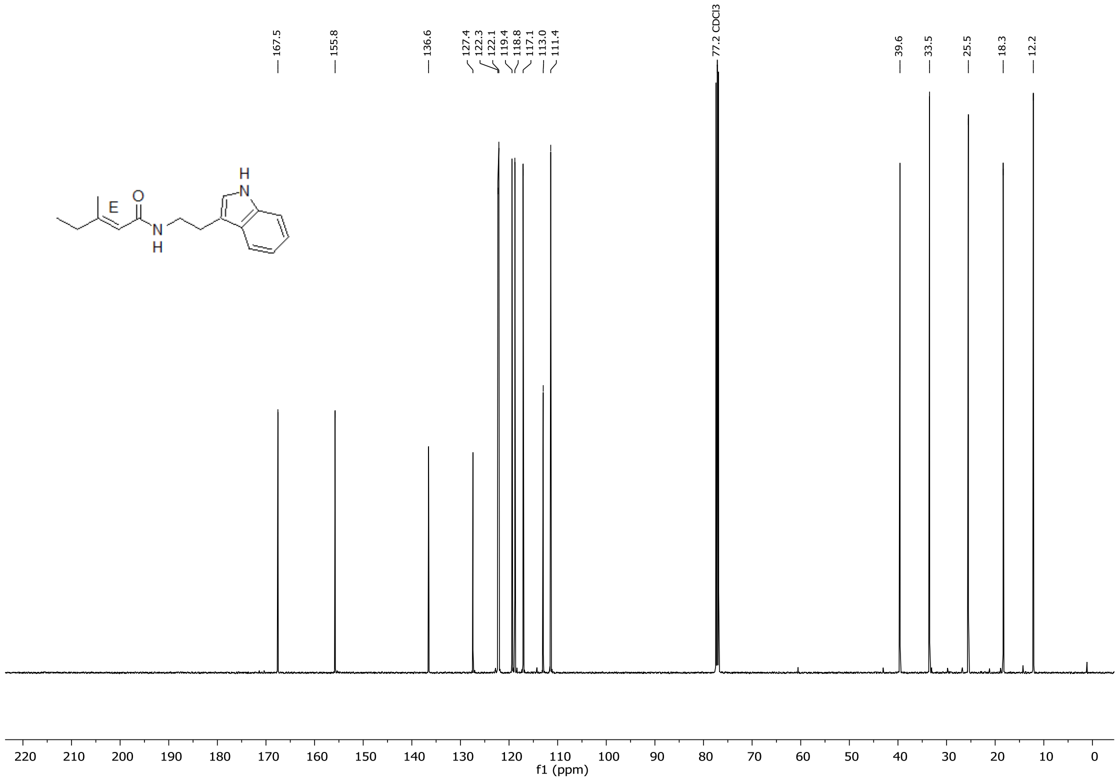

Figure S22: ^{13}C NMR (126 MHz, $CDCl_3$) of **11**.

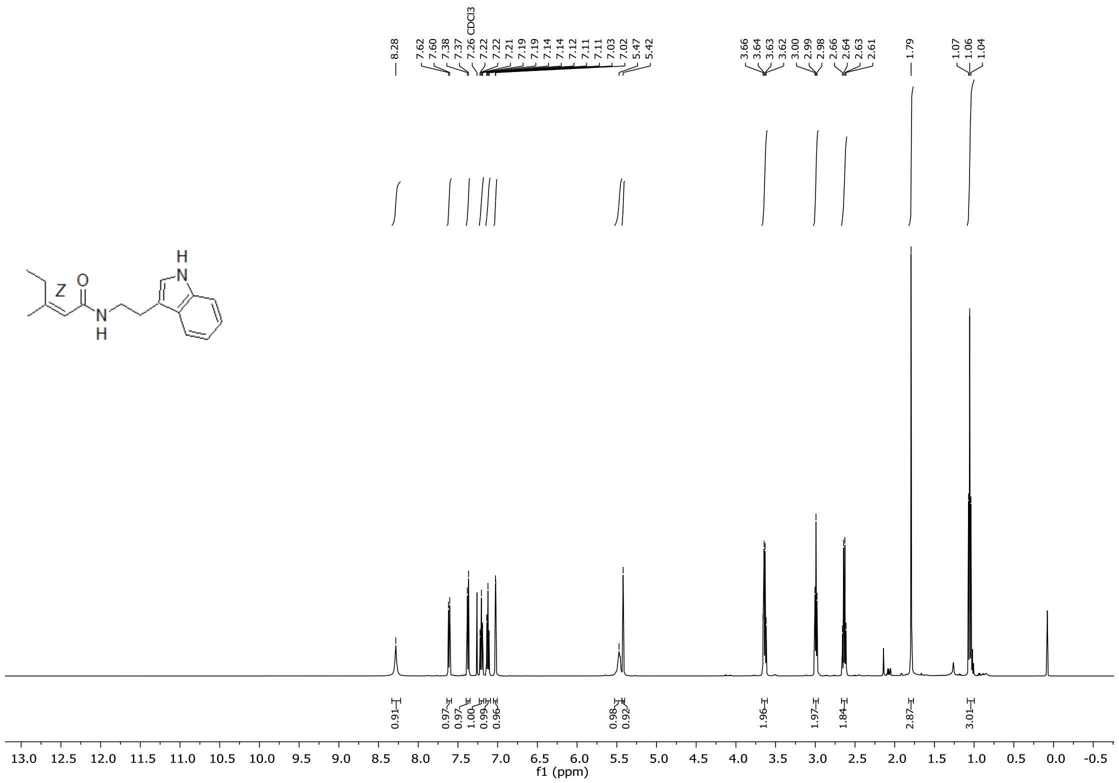

Figure S23: ^{1}H NMR spectra (500 MHz, $CDCl_3$) of **12**.

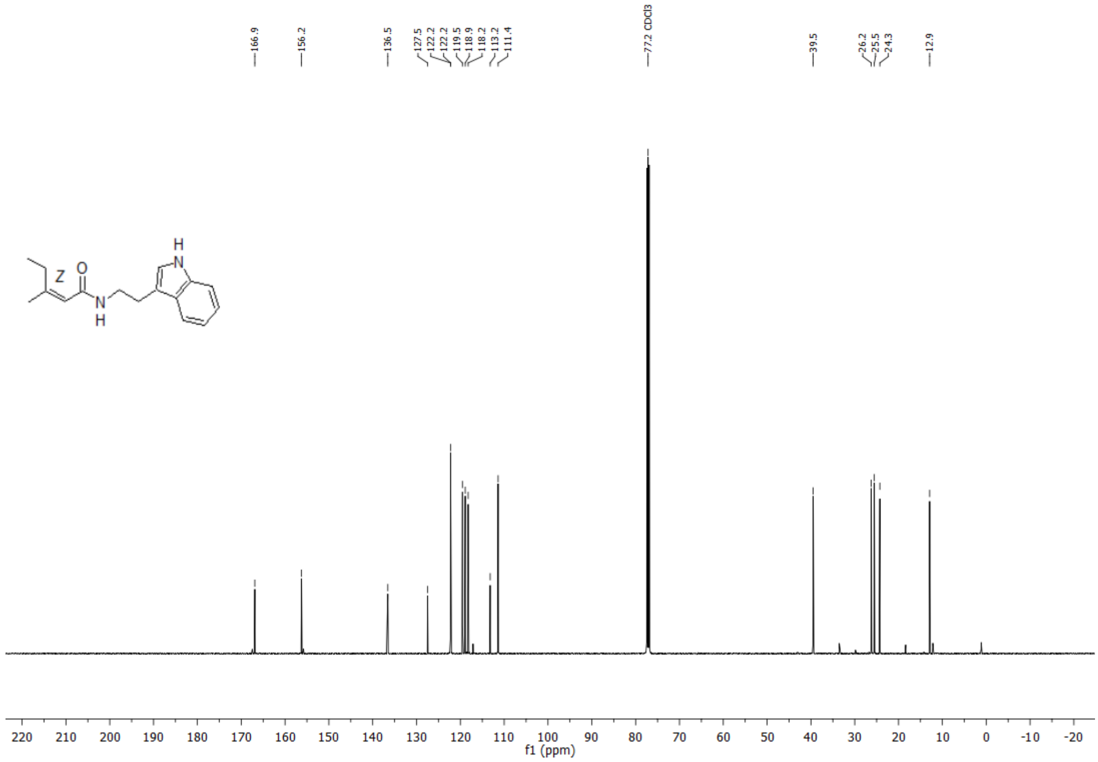

Figure S24: ^{13}C NMR (126 MHz, $CDCl_3$) of **12**.

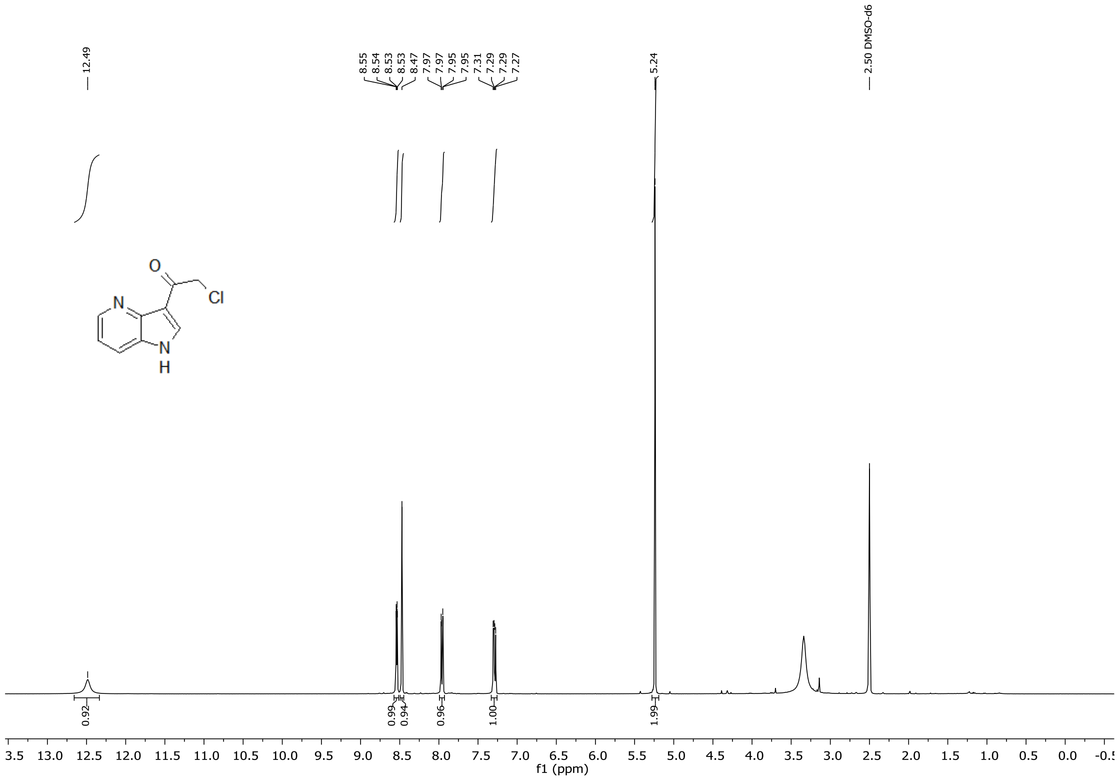

Figure S25: ^{1}H NMR spectra (400 MHz, DMSO-d_6) of **14**.

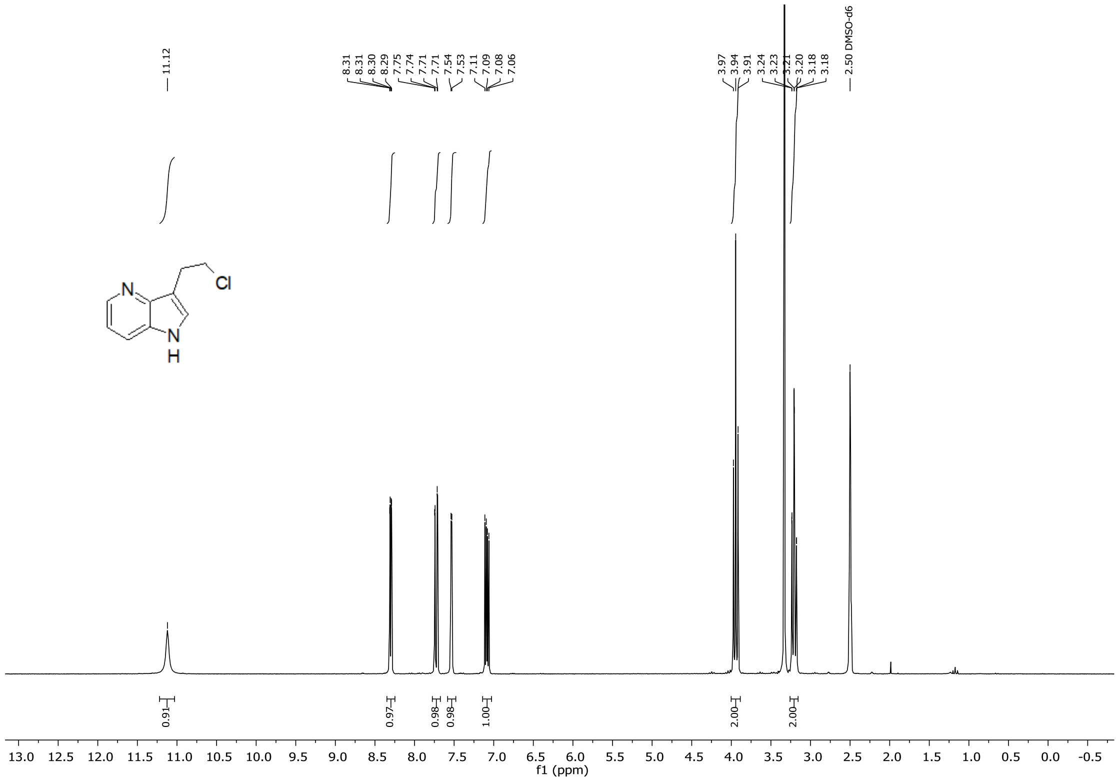

Figure S26: ^{1}H NMR spectra (250 MHz, DMSO-d_6) of **15**.

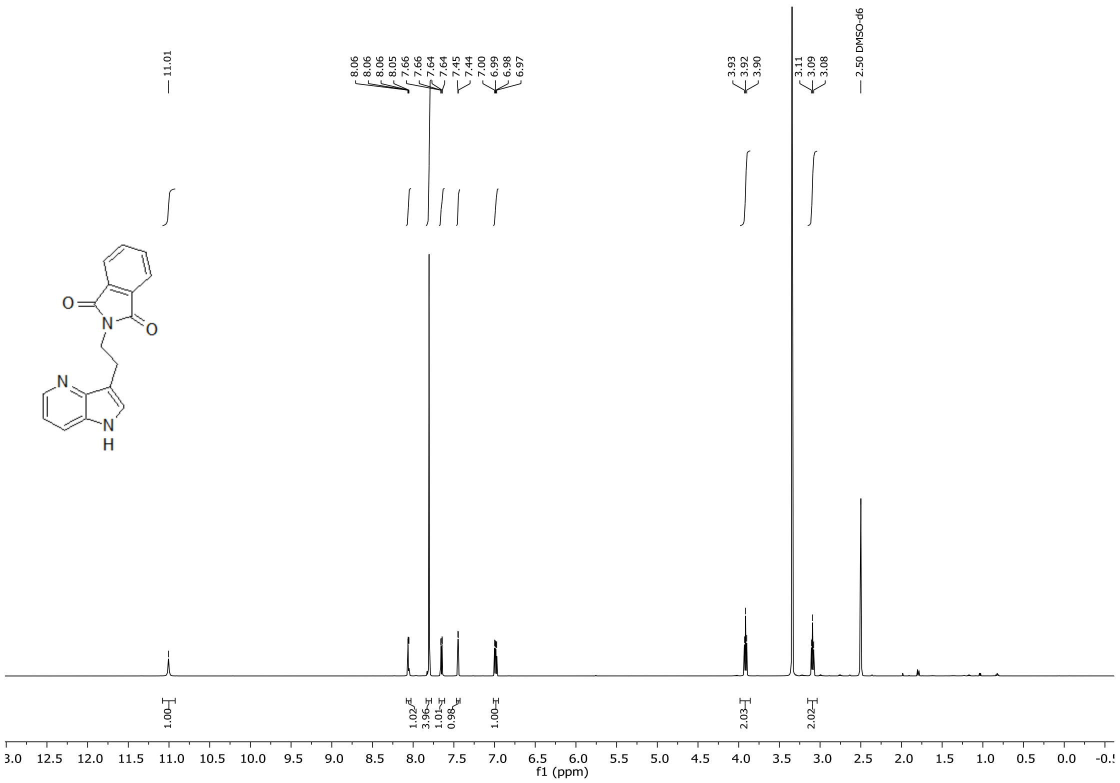

Figure S27: ^{1}H NMR spectra (500 MHz, DMSO-d_6) of **16**.

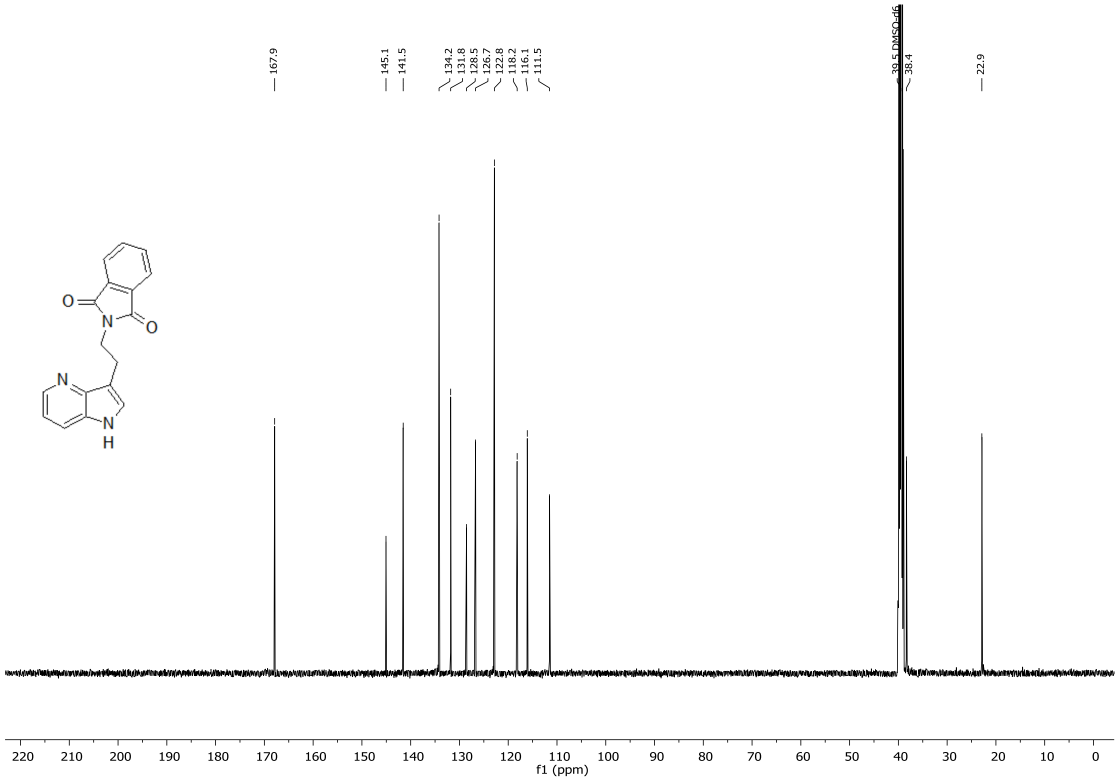

Figure S28: ^{13}C NMR (126 MHz, DMSO-d_6) of **16**.

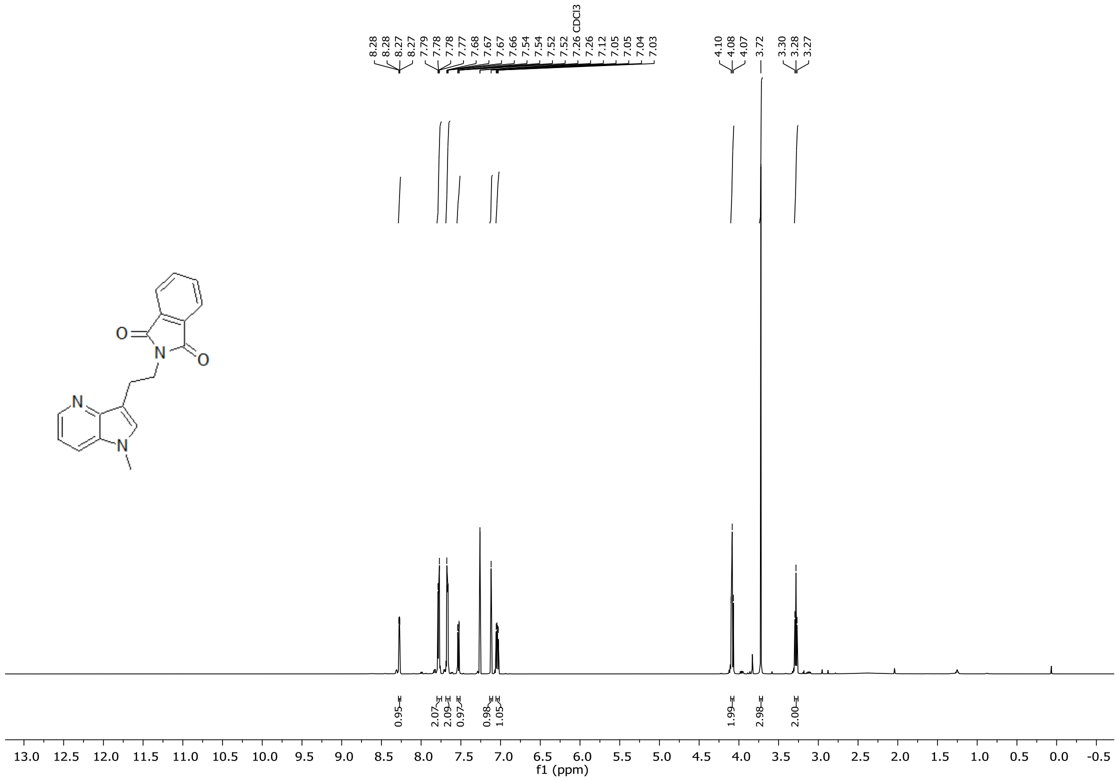

Figure S29: ^{1}H NMR spectra (500 MHz, $CDCl_3$) of **17**.

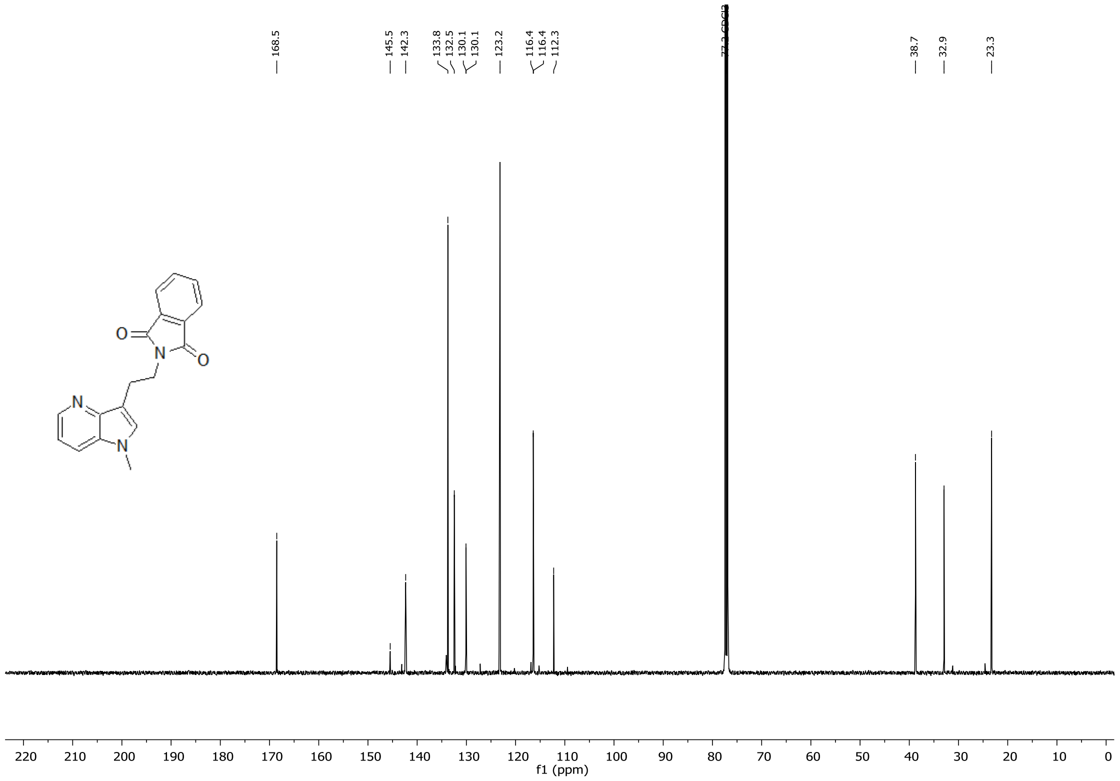

Figure S30: ^{13}C NMR (126 MHz, $CDCl_3$) of **17**.

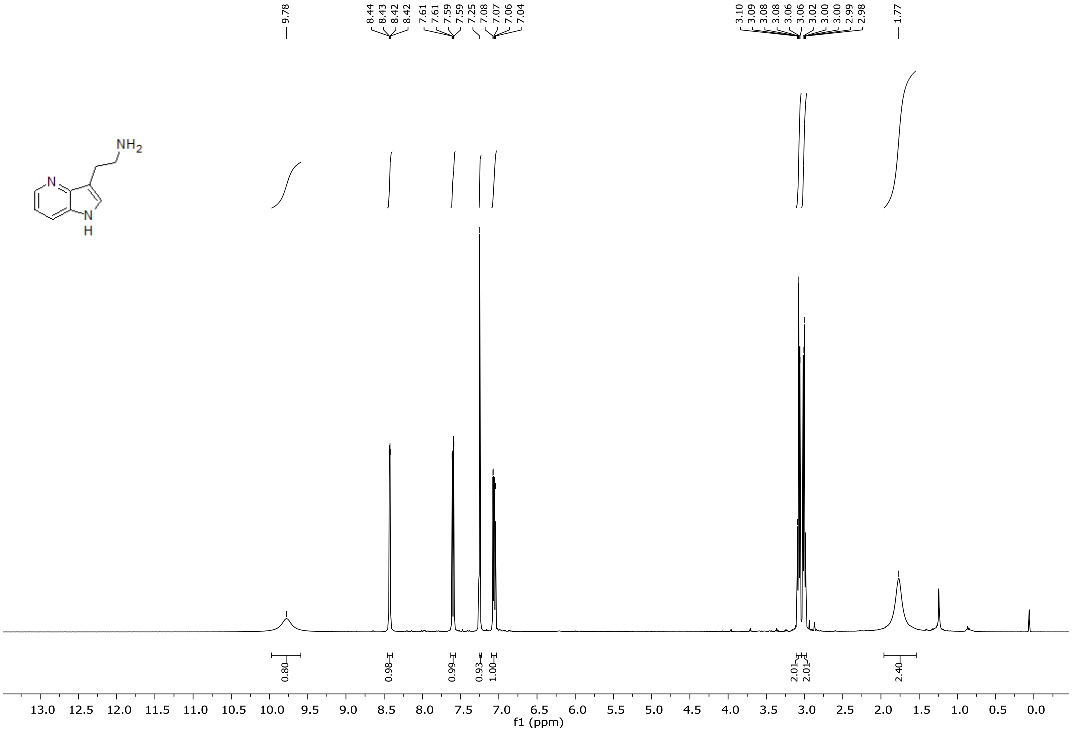

Figure S31: ^{1}H NMR spectra (400 MHz, $CDCl_3$) of **18**.

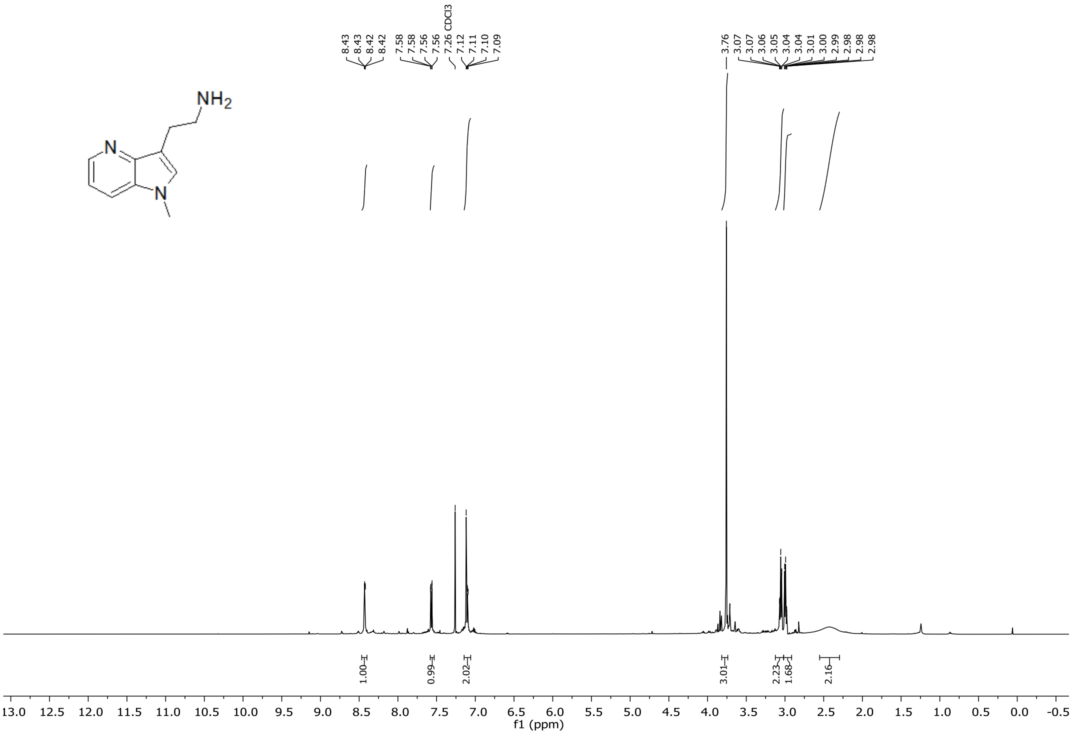

Figure S32: ^{1}H NMR spectra (500 MHz, $CDCl_3$) of **19**.

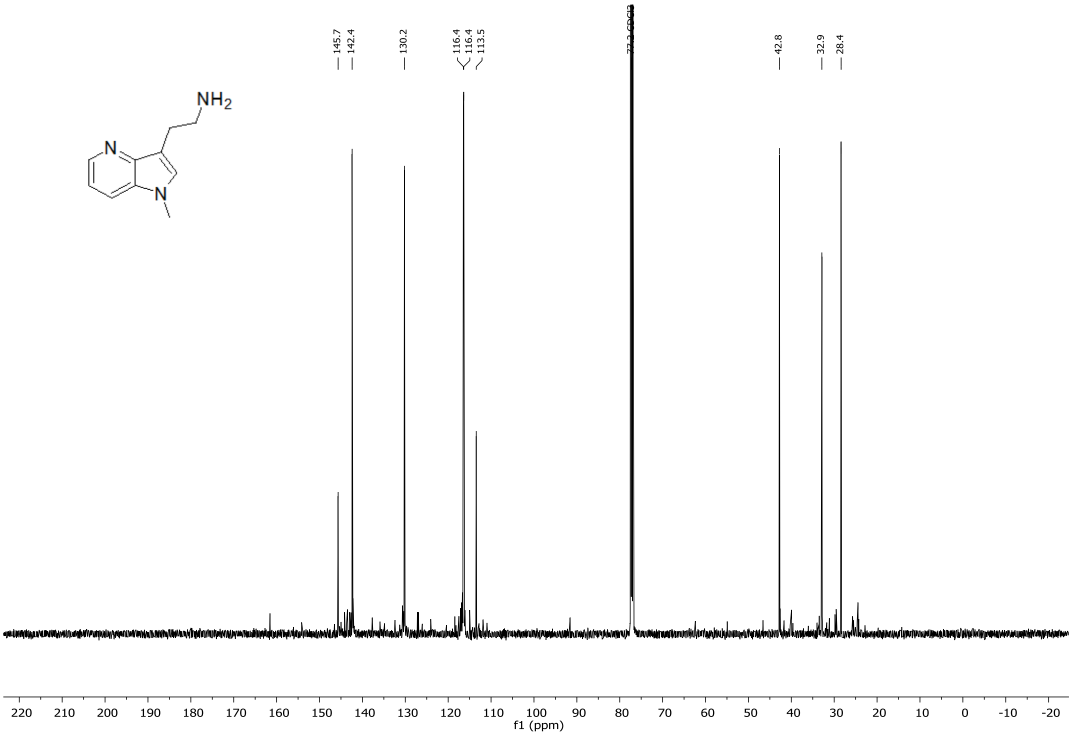

Figure S33: ^{13}C NMR (126 MHz, $CDCl_3$) of **19**.

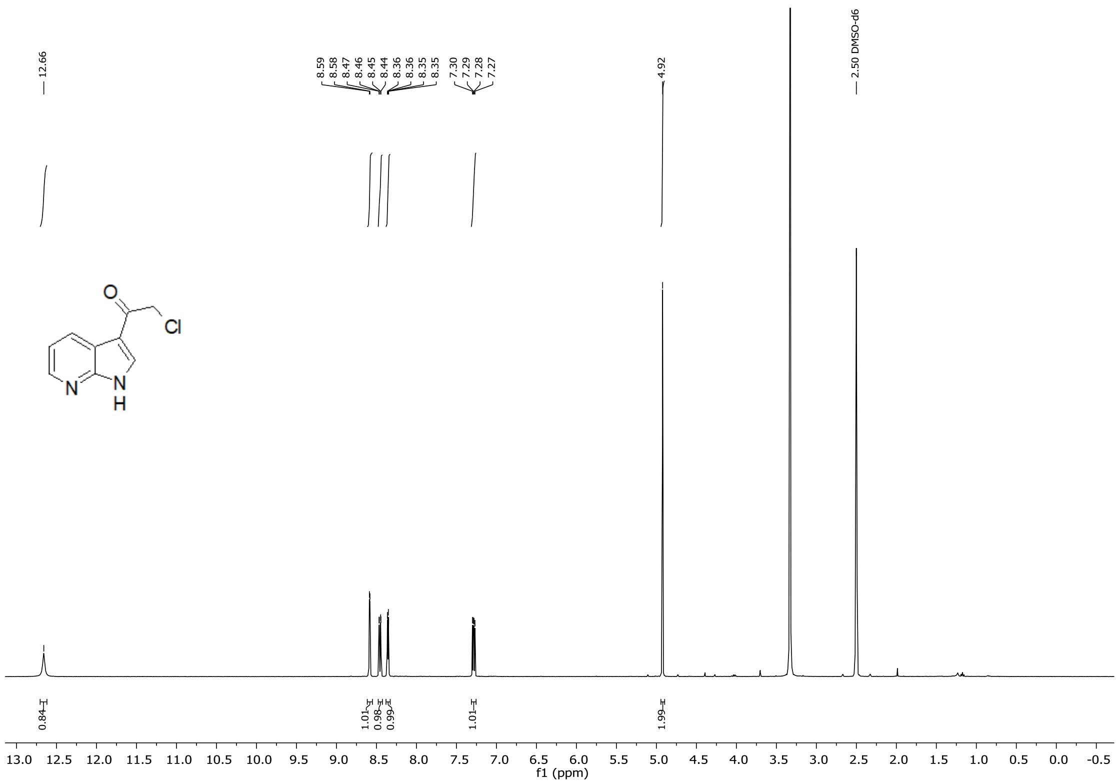

Figure S34: ^{1}H NMR spectra (400 MHz, DMSO-d_6) of **21**.

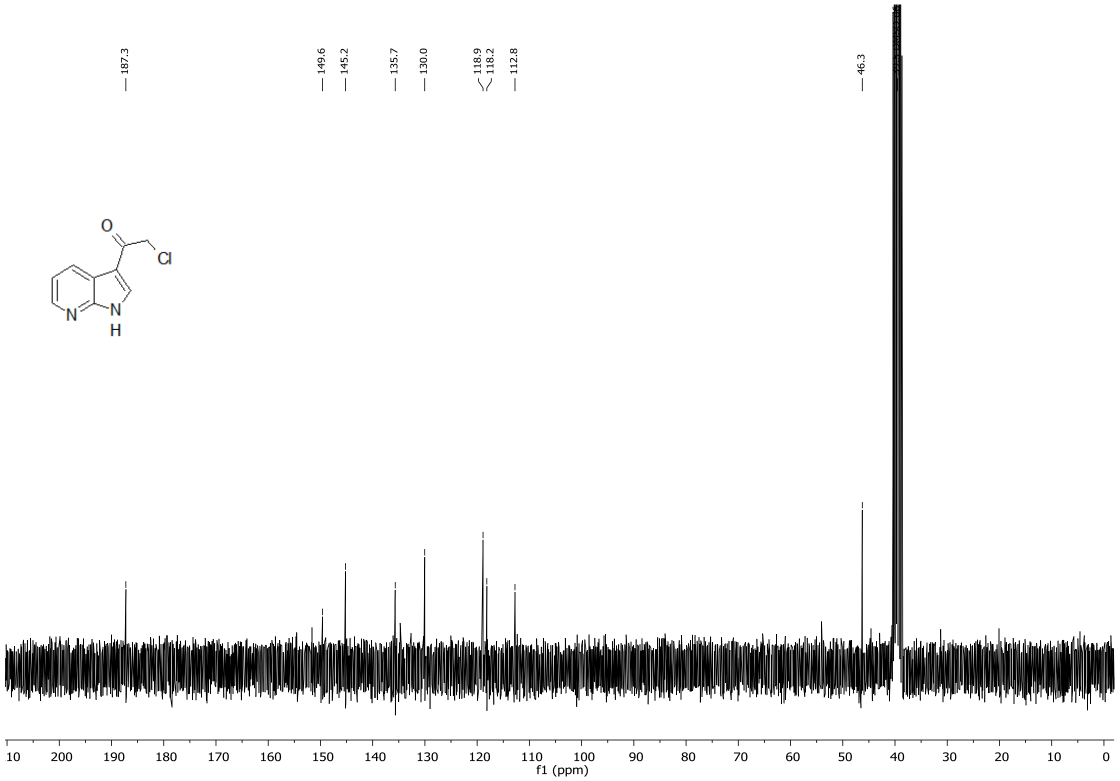

Figure S35: ^{13}C NMR spectra (75 MHz, DMSO-d_6) of **21**.

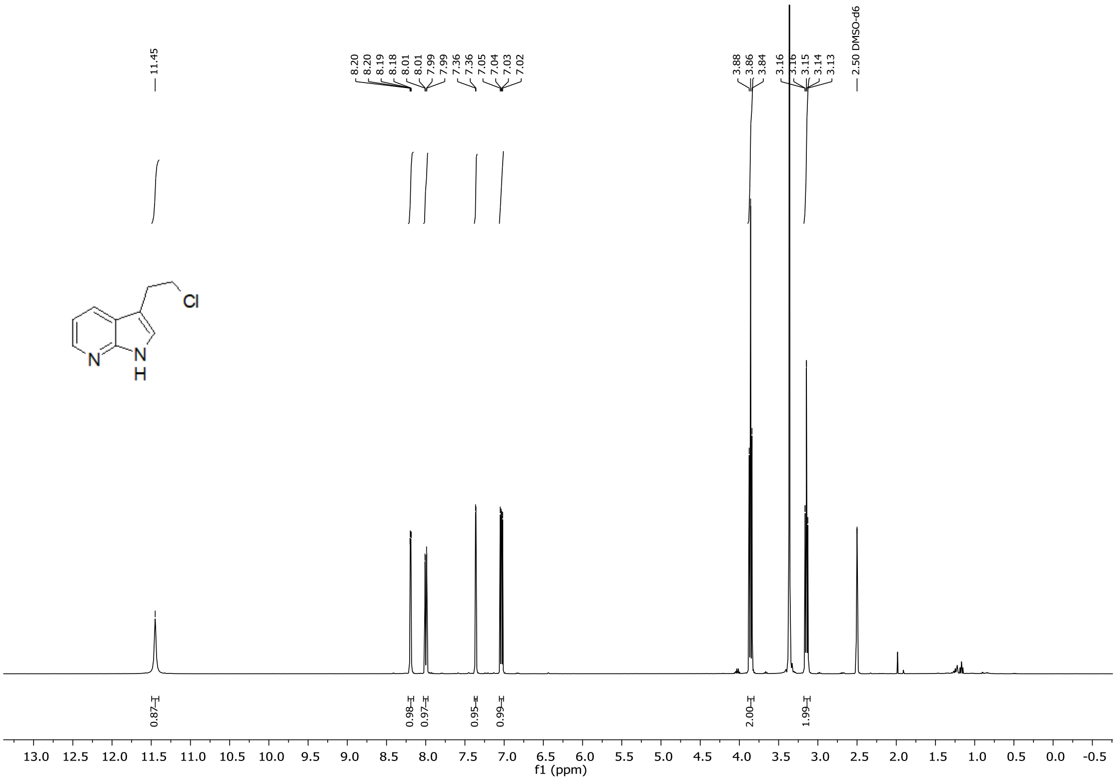

Figure S36: ^{1}H NMR spectra (250 MHz, DMSO-d_6) of **22**.

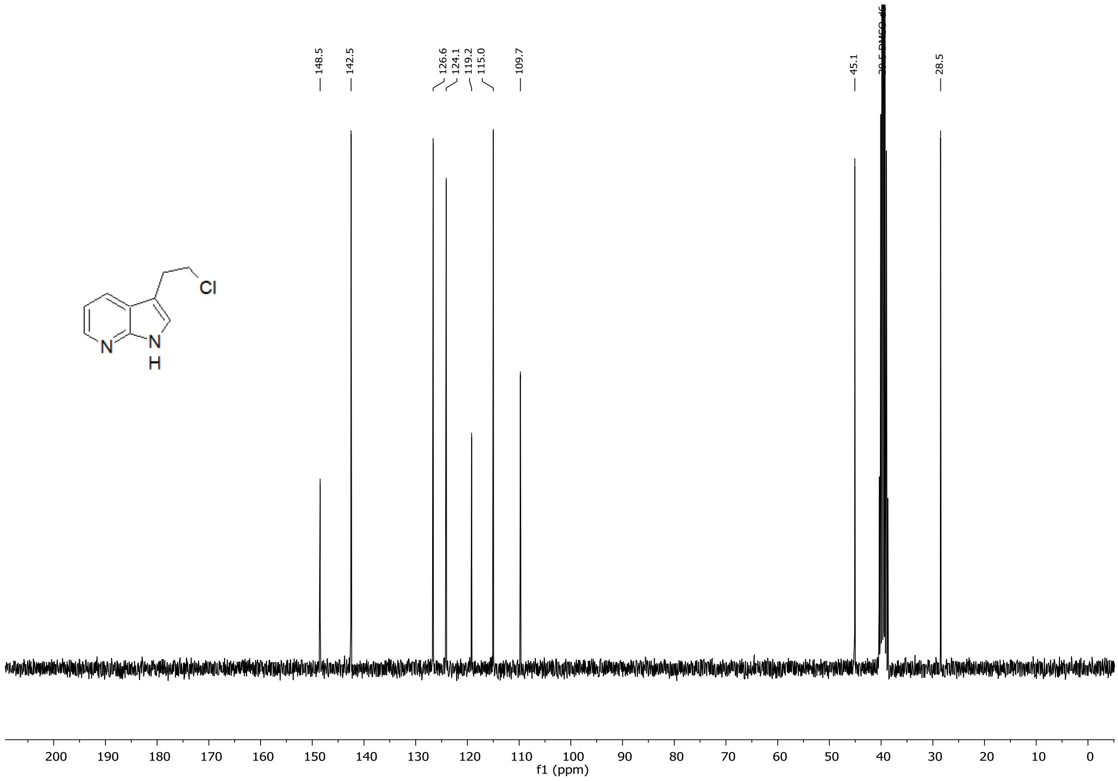

Figure S37: ^{13}C NMR (75 MHz, DMSO-d_6) of **22**.

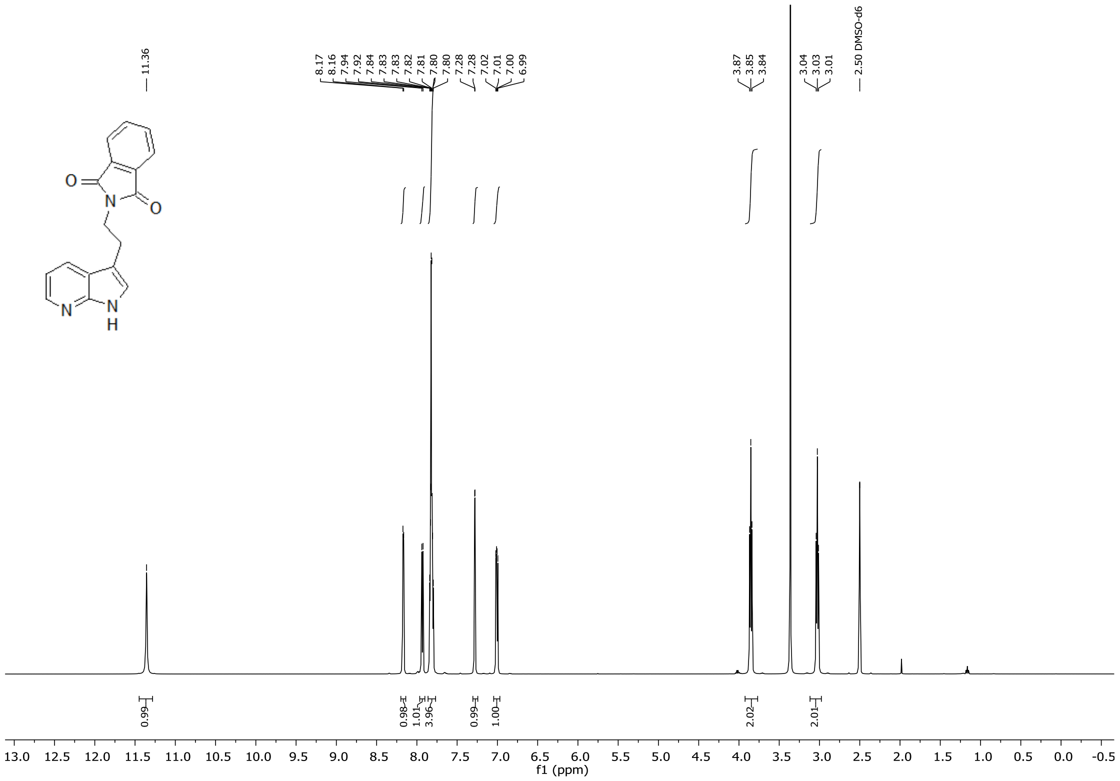

Figure S38: ^{1}H NMR spectra (500 MHz, DMSO-d_6) of **23**.

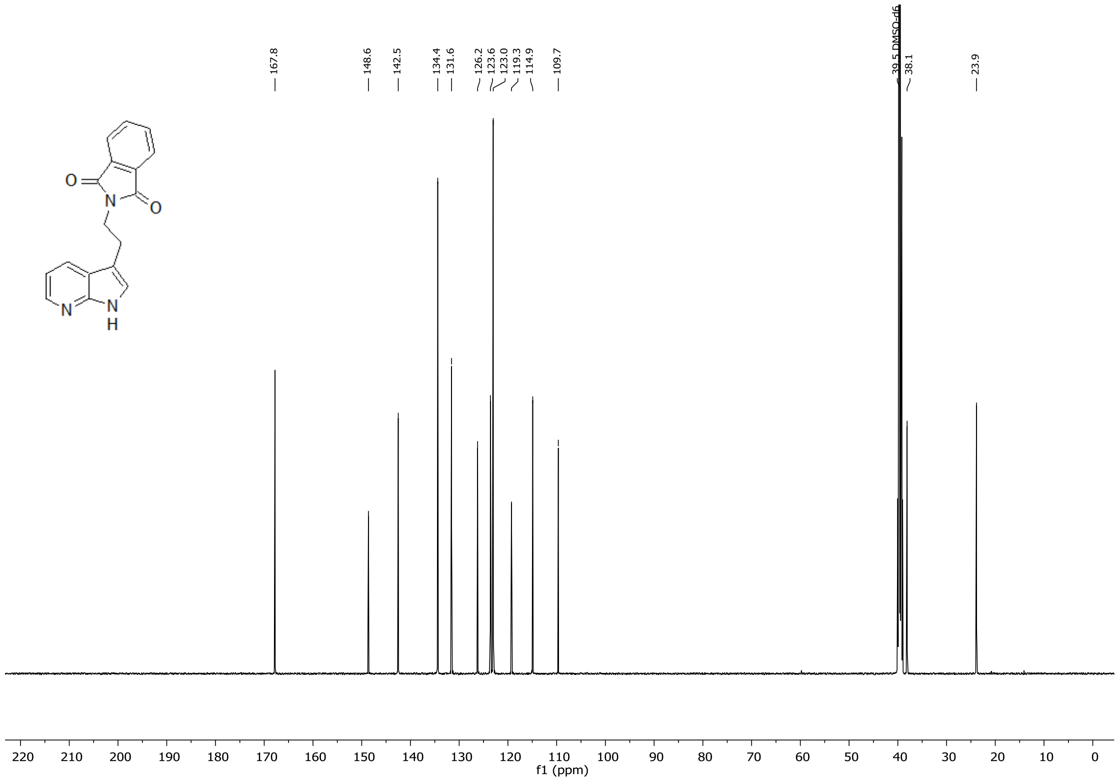

Figure S39: ^{13}C NMR (126 MHz, DMSO-d_6) of **23**.

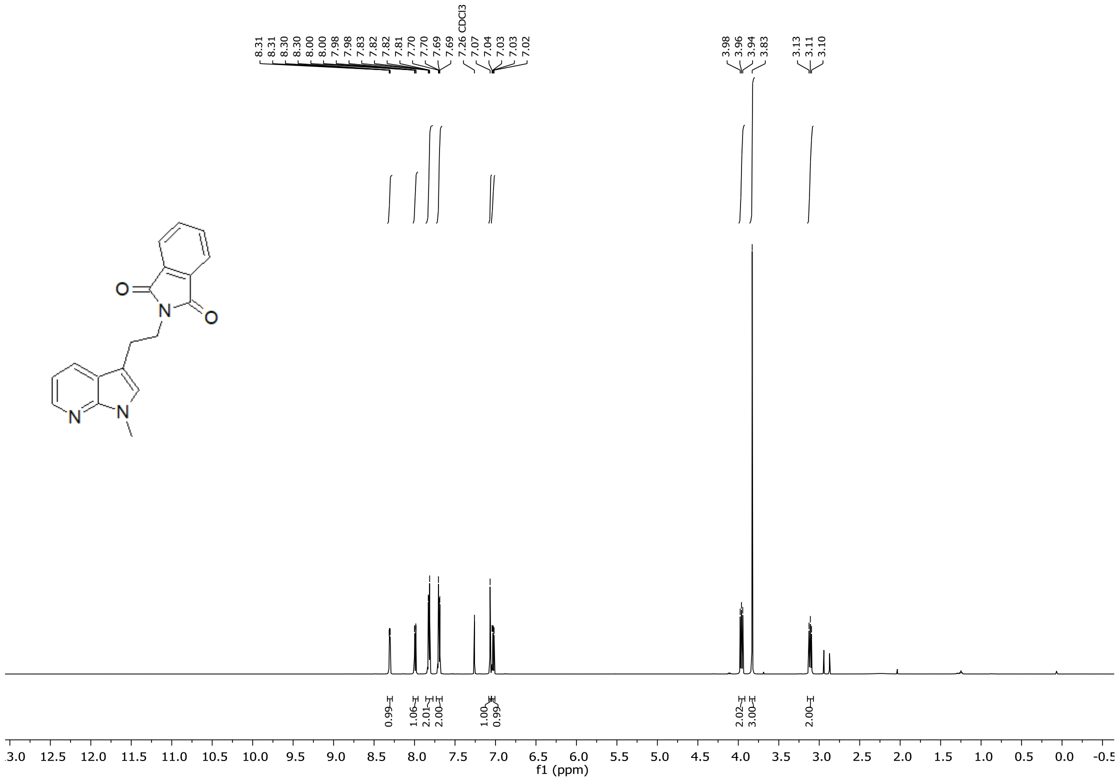

Figure S40: ^{1}H NMR spectra (500 MHz, $CDCl_3$) of **24**.

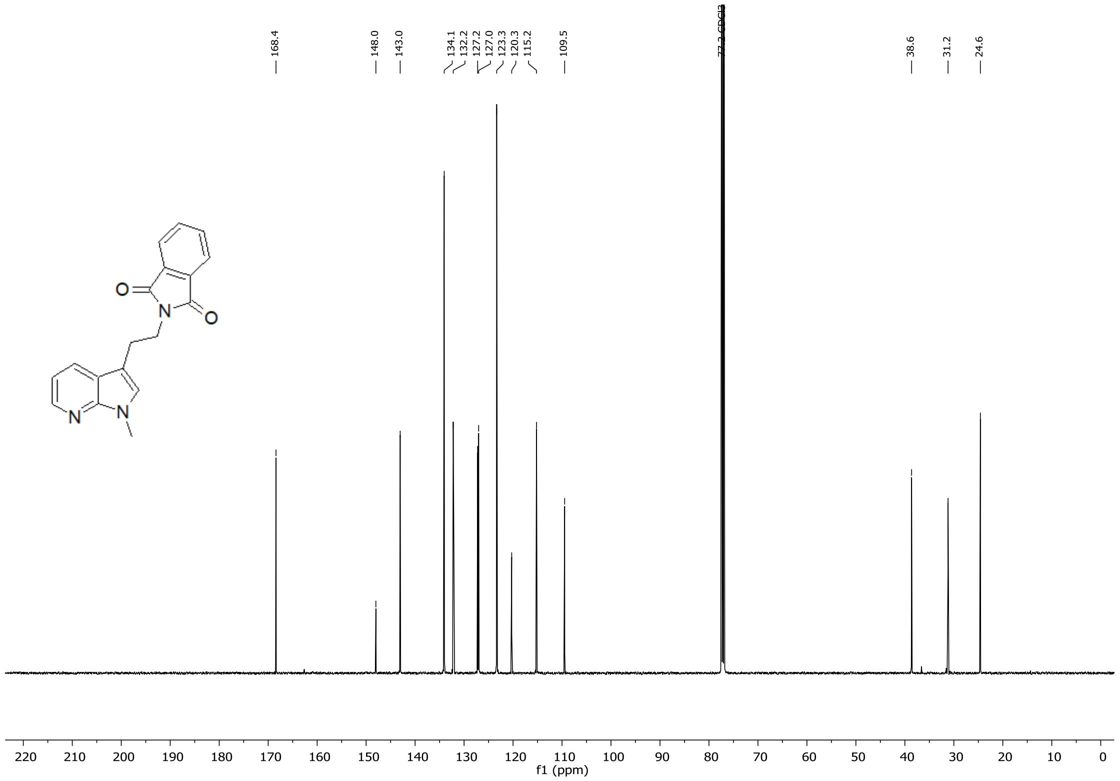

Figure S41: ^{13}C NMR (126 MHz, $CDCl_3$) of **24**.

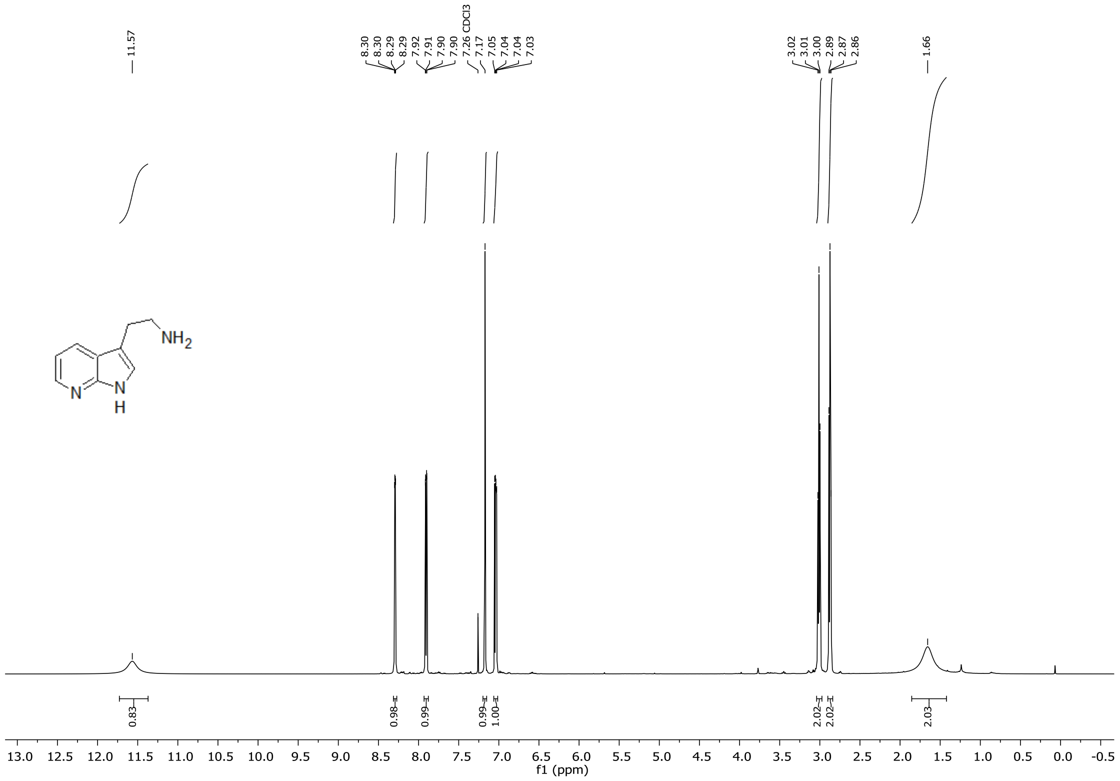

Figure S42: ^{1}H NMR spectra (500 MHz, $CDCl_3$) of **25**.

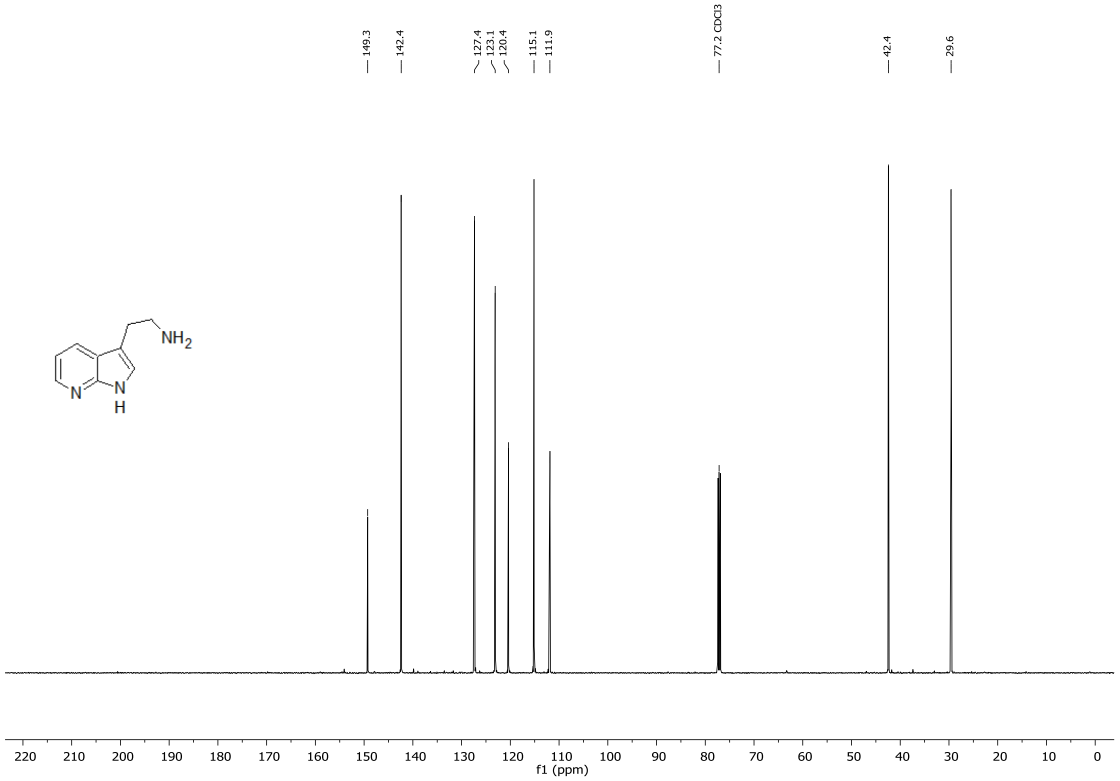

Figure S43: ^{13}C NMR (126 MHz, $CDCl_3$) of **25**.

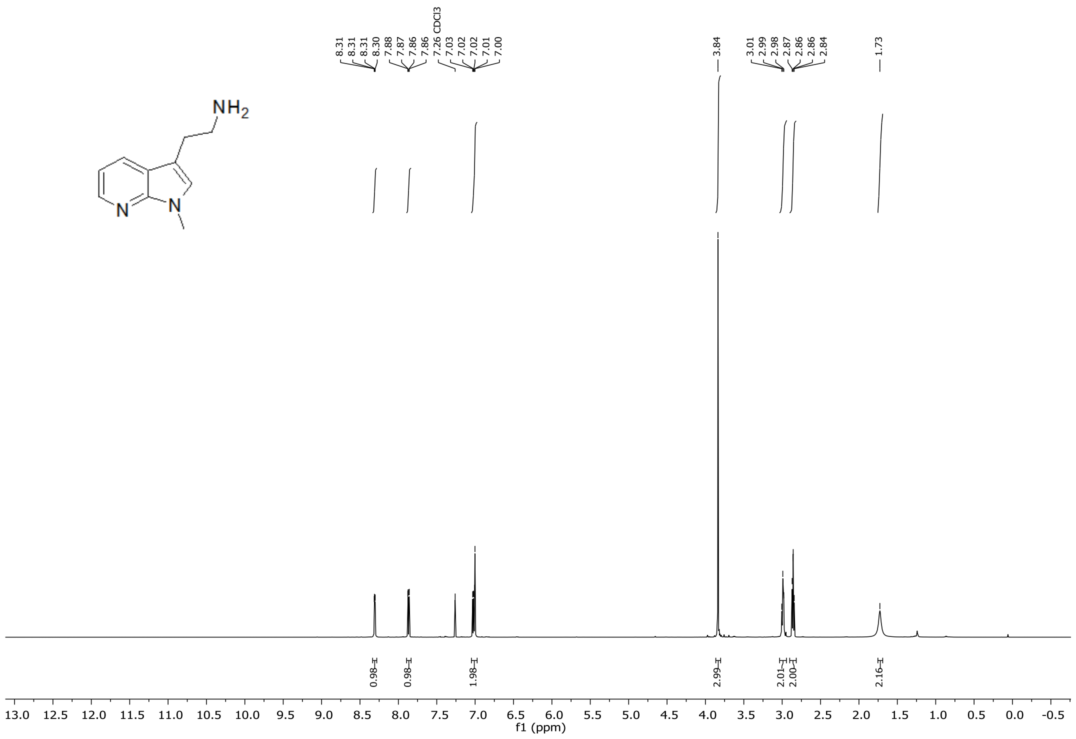

Figure S44: ^{1}H NMR spectra (500 MHz, $CDCl_3$) of **26**.

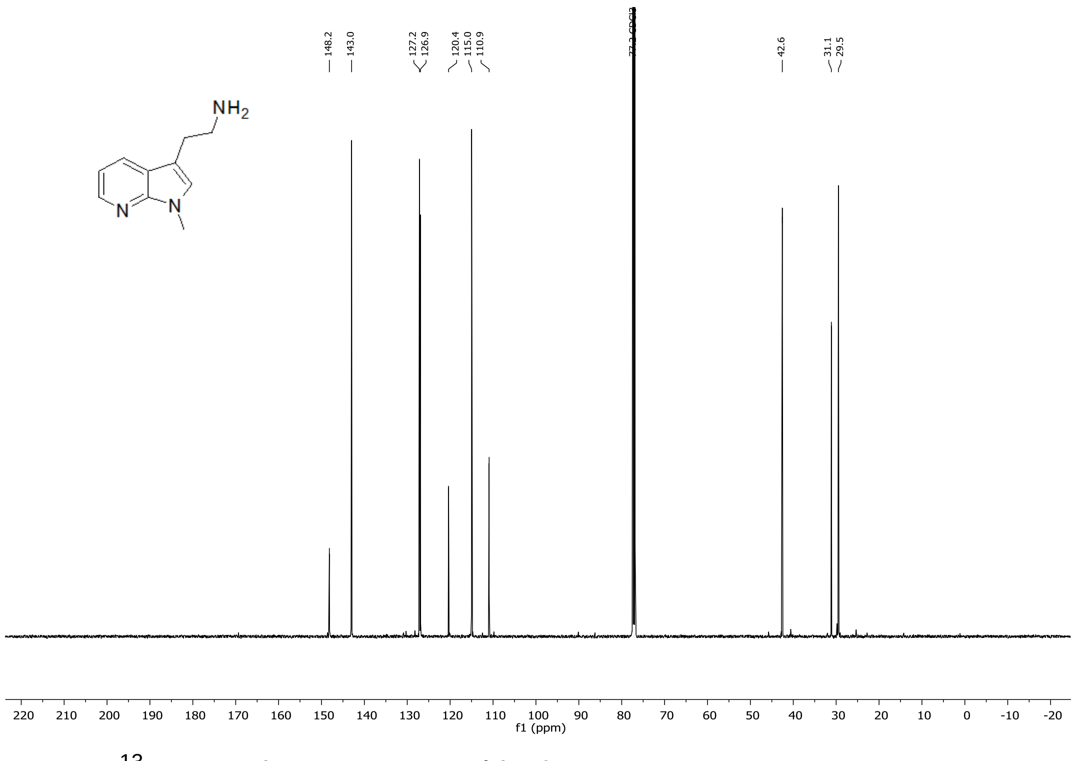

Figure S45: ^{13}C NMR (126 MHz, $CDCl_3$) of **26**.

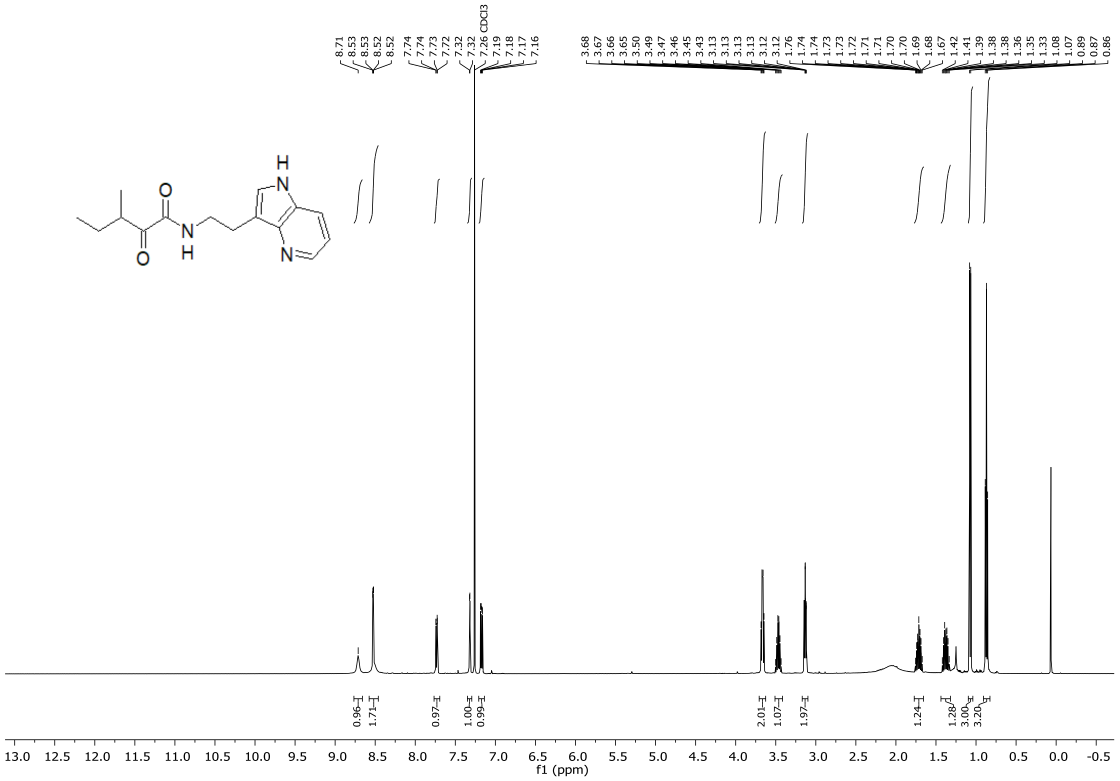

Figure S46: ^{1}H NMR spectra (500 MHz, $CDCl_3$) of **27**.

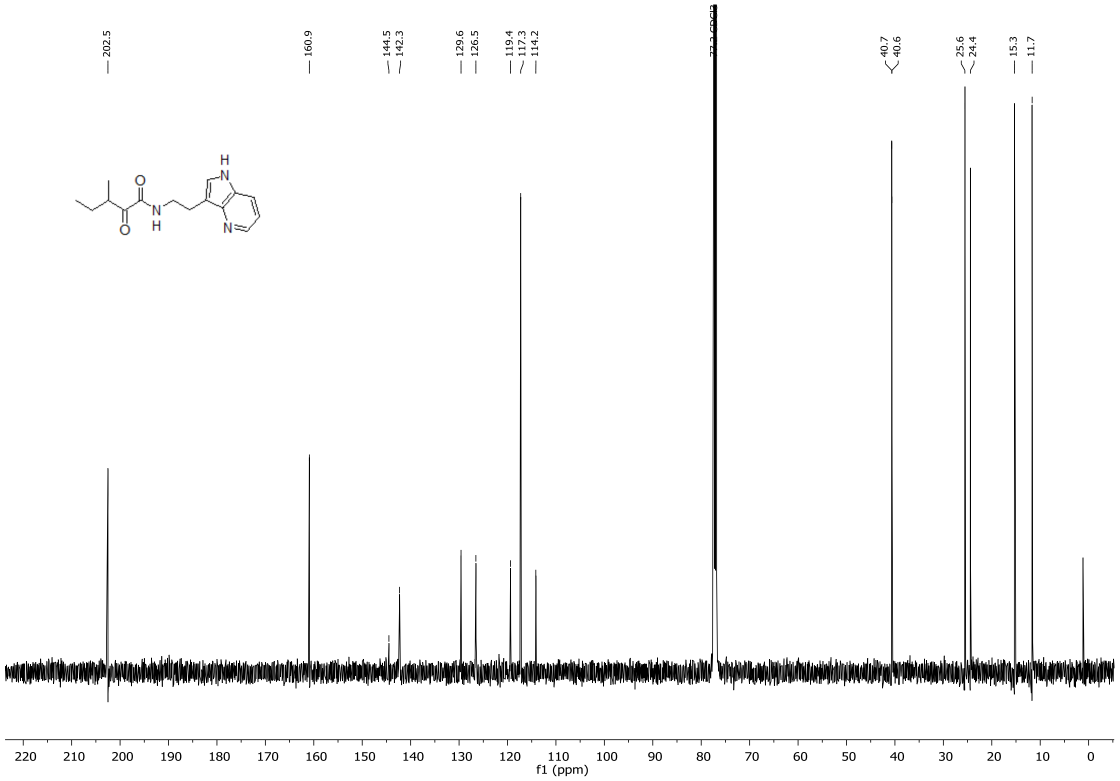

Figure S47: ^{13}C NMR (126 MHz, $CDCl_3$) of **27**.

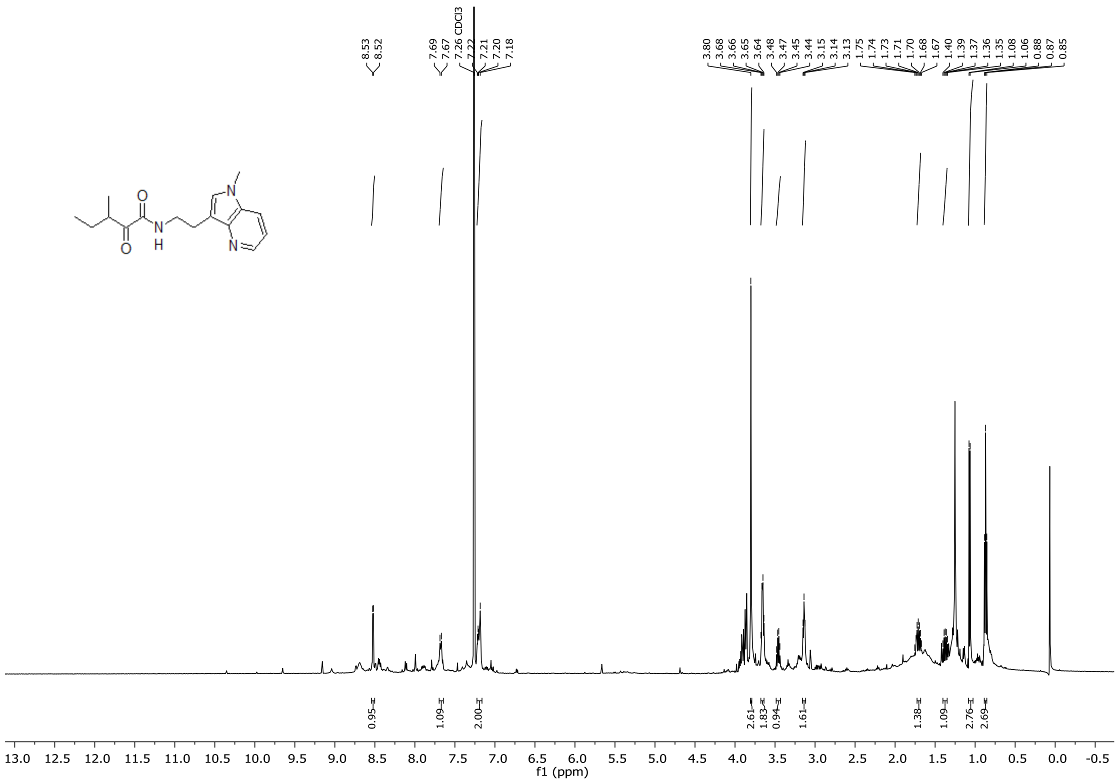

Figure S48: ^{1}H NMR spectra (500 MHz, $CDCl_3$) of **28**.

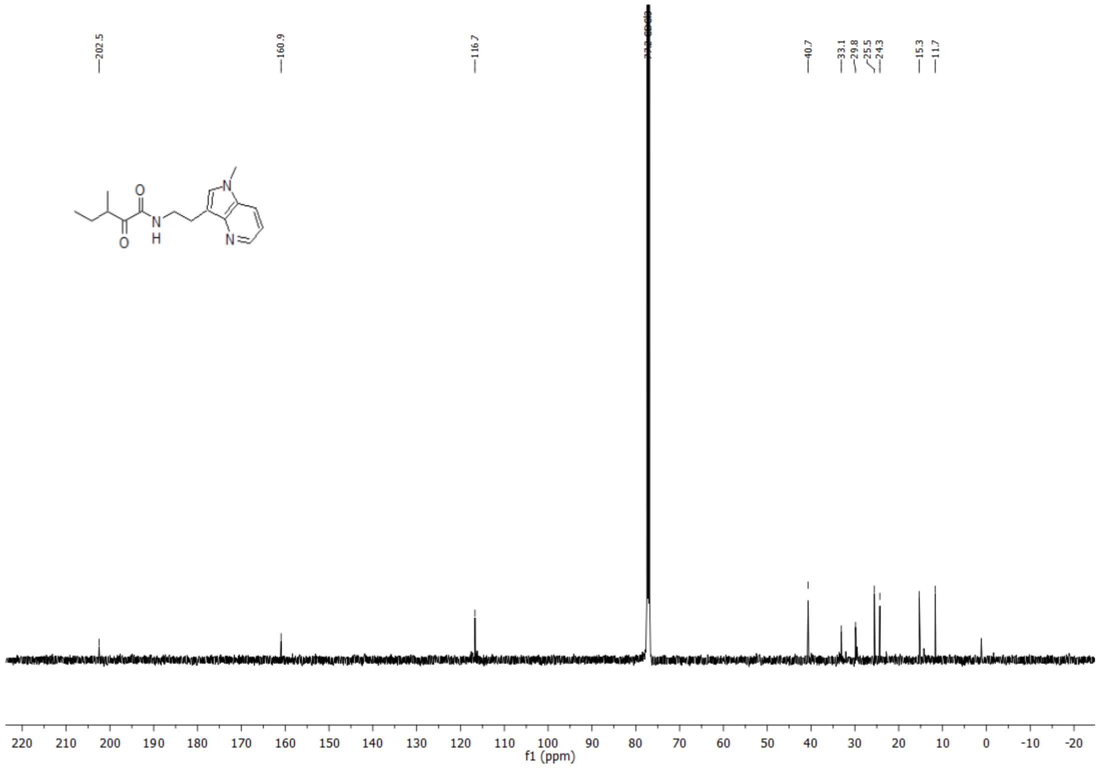

Figure S49: ^{13}C NMR (126 MHz, $CDCl_3$) of **28**.

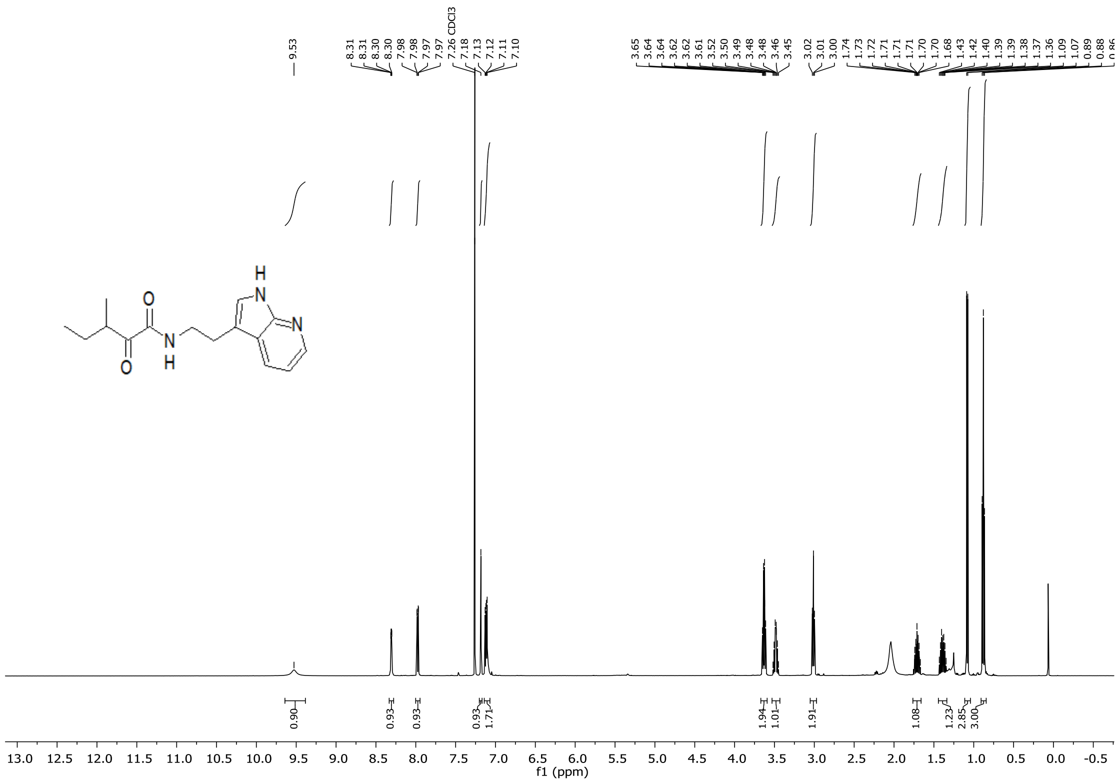

Figure S50: ^{1}H NMR spectra (500 MHz, $CDCl_3$) of **29**.

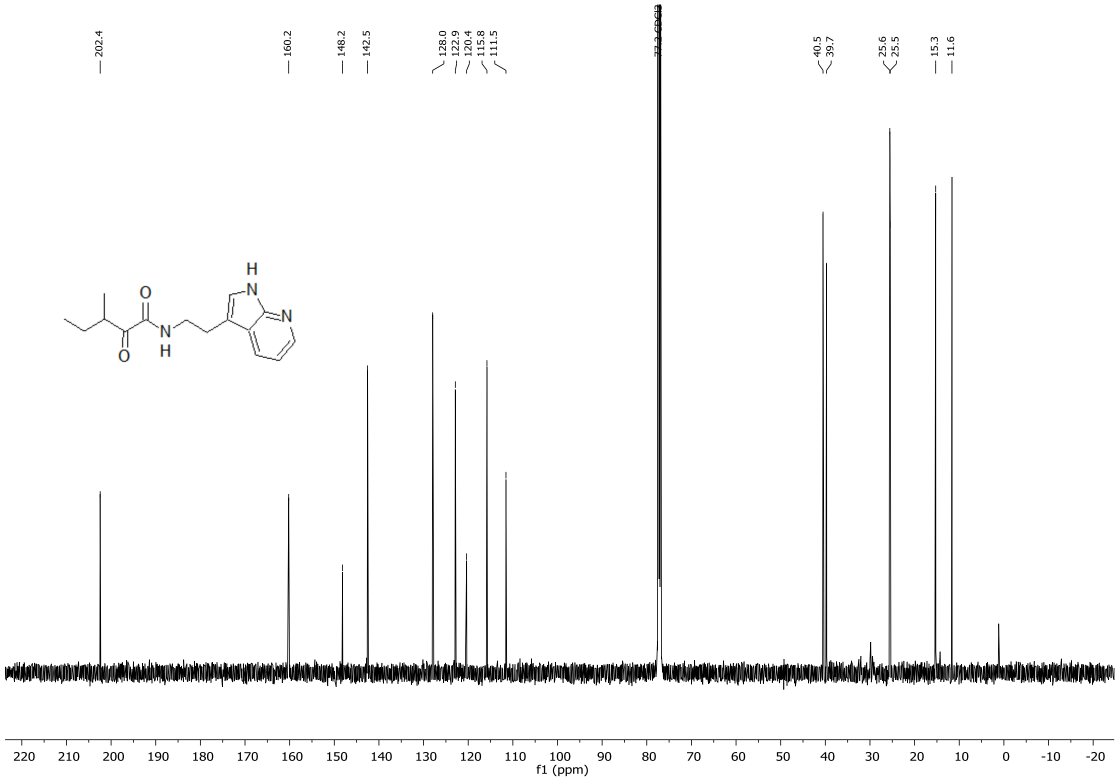

Figure S51: ^{13}C NMR (126 MHz, $CDCl_3$) of **29**.

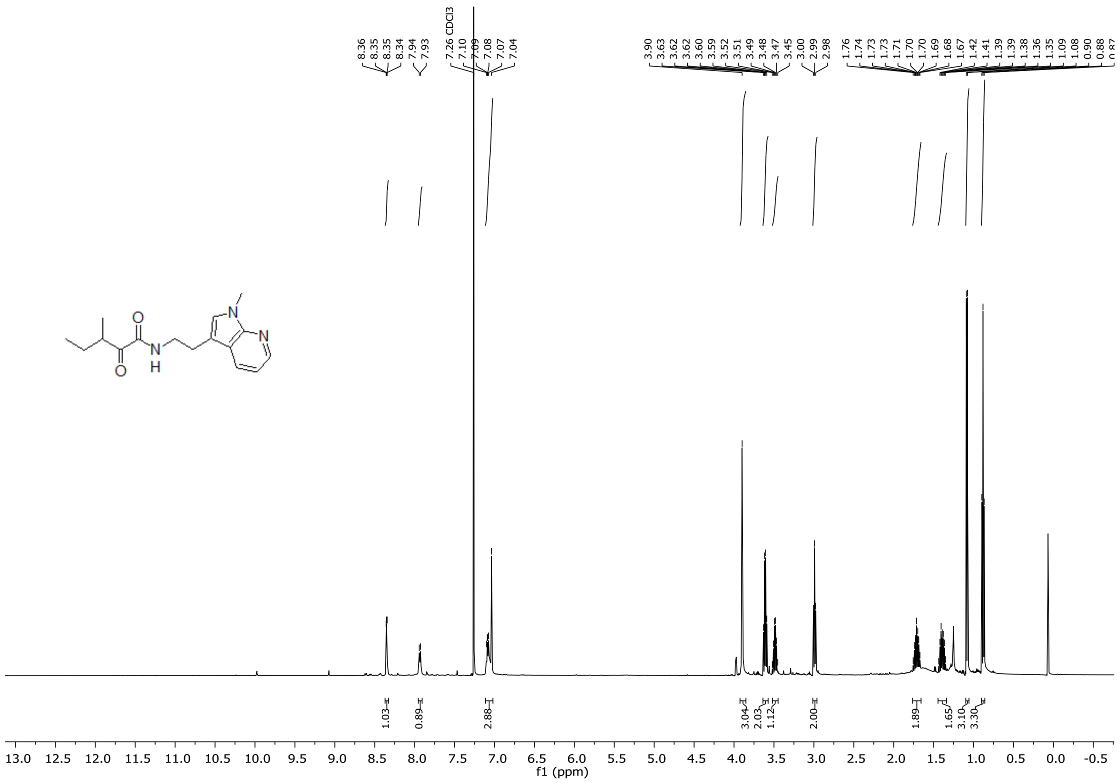

Figure S52: ^{1}H NMR spectra (500 MHz, $CDCl_3$) of **30**.

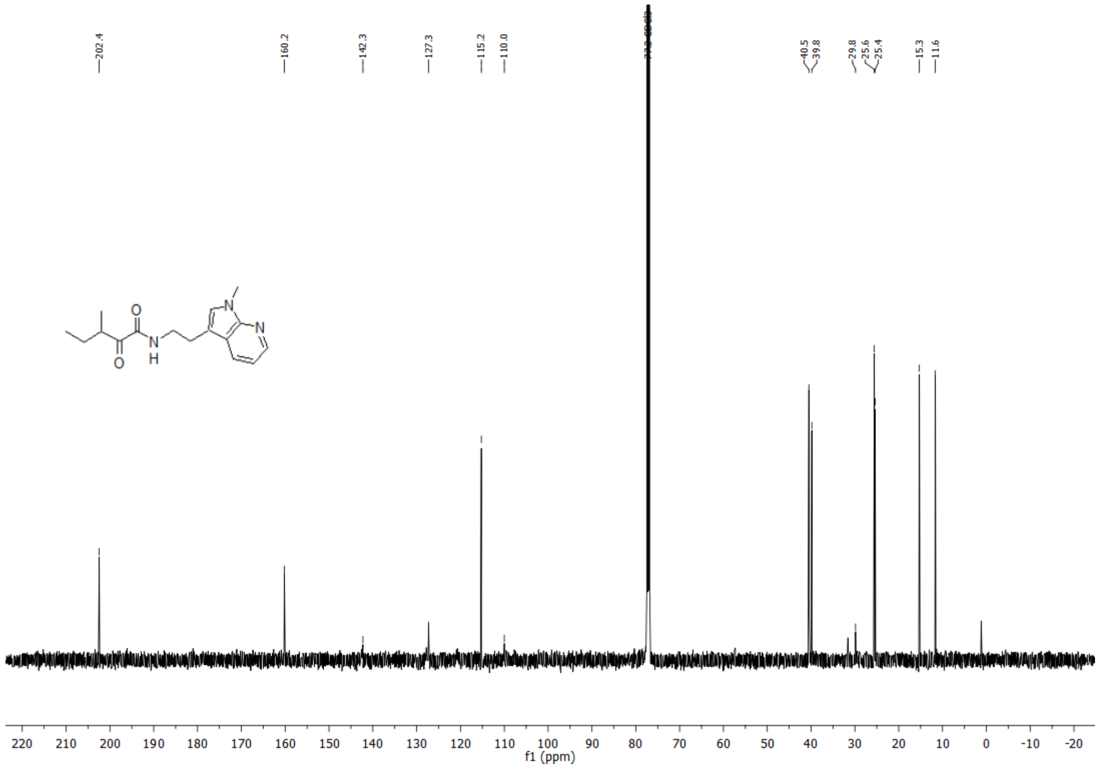

Figure S53: ^{13}C NMR (126 MHz, $CDCl_3$) of **30**.

References

(1) Dannhardt, G.; Kramb, J.-P.; Plutizki, S. EP 2343291A1, 09179986.6 **2009**.
